# Defects of Vps15 in skeletal muscles lead to autophagic vacuolar myopathy and lysosomal disease

**DOI:** 10.1002/emmm.201202057

**Published:** 2013-04-30

**Authors:** Ivan Nemazanyy, Bert Blaauw, Cecilia Paolini, Catherine Caillaud, Feliciano Protasi, Amelie Mueller, Tassula Proikas-Cezanne, Ryan C Russell, Kun-Liang Guan, Ichizo Nishino, Marco Sandri, Mario Pende, Ganna Panasyuk

**Affiliations:** 1 Inserm, U845Paris, France; 2 Université Paris Descartes, Faculté de Médecine, UMRS-845Paris, France; 3 Venetian Institute of Molecular MedicinePadova, Italy; 4 Dipartimento Neuroscienze Imaging, Università “G. d'Annunzio”Chieti, France; 5 Eberhard Karls University TuebingenTuebingen, Germany; 6 Department of Pharmacology and Moores Cancer Center, University of CaliforniaSan Diego, La Jolla, CA, USA; 7 National Institute of Neuroscience, National Center of Neurology and PsychiatryTokyo, Japan

**Keywords:** autophagy, human disease, lysosomal storage disease, mouse model, mTOR signalling

## Abstract

The complex of Vacuolar Protein Sorting 34 and 15 (Vps34 and Vps15) has Class III phosphatidylinositol 3-kinase activity and putative roles in nutrient sensing, mammalian Target Of Rapamycin (mTOR) activation by amino acids, cell growth, vesicular trafficking and autophagy. Contrary to expectations, here we show that *Vps15*-deficient mouse tissues are competent for LC3-positive autophagosome formation and maintain mTOR activation. However, an impaired lysosomal function in mutant cells is traced by accumulation of adaptor protein p62, LC3 and Lamp2 positive vesicles, which can be reverted to normal levels after ectopic overexpression of Vps15. Mice lacking Vps15 in skeletal muscles, develop a severe myopathy. Distinct from the autophagy deficient *Atg7*^−/−^ mutants, pathognomonic morphological hallmarks of autophagic vacuolar myopathy (AVM) are observed in *Vps15*^−/−^ mutants, including elevated creatine kinase plasma levels, accumulation of autophagosomes, glycogen and sarcolemmal features within the fibres. Importantly, Vps34/Vps15 overexpression in myoblasts of Danon AVM disease patients alleviates the glycogen accumulation. Thus, the activity of the Vps34/Vps15 complex is critical in disease conditions such as AVMs, and possibly a variety of other lysosomal storage diseases.

## INTRODUCTION

The *vps34* and *vps15* genes were initially identified in yeast screens for mutants defective in protein localization, sorting and processing to the vacuole, the equivalent of mammalian lysosome (Banta et al, 1988; Robinson et al, 1988). *vps34* and *vps15* mutant yeast strains display severe defects in the sorting and delivery of soluble hydrolases, including carboxypeptidase Y, from the late Golgi to the vacuole. Another striking phenotype of these mutants is impaired macroautophagy (hereafter referred to as autophagy), a major process in response to starvation conditions (Kihara et al, 2001). Autophagy begins with the formation of an autophagosome, a double membrane structure that engulfs parts of the cytoplasm and whole organelles, and ultimately fuses with a lysosome to enable degradation of the enclosed material (Mehrpour et al, 2010). Vps34 and Vps15 proteins are obligate partners acting in a complex. Vps34 is a Class III phosphatidylinositol 3-kinase (PI3K) converting phosphatidylinositol (PI) to phosphatidylinositol 3-phosphate (PI3P) (Schu et al, 1993), while Vps15 is a regulatory subunit with putative serine/threonine kinase activity and is required for Vps34 stability and activation (Schu et al, 1993). The conservation of *Vps15* and *Vps34* genes from yeast to mammals suggests their evolutionary conserved role in essential cellular functions, prompting the phenotypic analysis of mammalian mutants.

The different functions of Vps34/Vps15 appear to be mediated by specific complexes. Atg6 and Vps38 are additional partners of Vps34/Vps15 in a protein complex for carboxypeptidase Y sorting, while a complex containing Vps34, Vps15, Atg6 and Atg14, but not Vps38, is required in yeast for autophagy (Kihara et al, 2001). In mammals at least three different Vps15/Vps34 complexes regulate different stages of autophagy. Each complex contains Beclin 1, the mammalian orthologue of Atg6. An Atg14-like (Atg14L) containing complex stimulates autophagosome formation, while ultraviolet radiation resistance-associated gene protein (UVRAG, the putative orthologue of Vps38) belongs to a complex that enhances or suppresses the maturation of autophagosome and endosome depending on the presence of the Rubicon protein (Matsunaga et al, 2009; Zhong et al, 2009). The existence of additional functions carried by distinct complexes has been postulated, as RNAi against Vps34 and Vps15 down-regulates mammalian Target Of Rapamycin (mTOR, now officially named mechanistic Target Of Rapamycin) activity upon amino acid stimulation in human cells (Byfield et al, 2005; Nobukuni et al, 2005; Yoon et al, 2011). Since mTOR is a known negative regulator of autophagy (Jung et al, 2010), these findings would therefore imply that different Vps15/Vps34 complexes might be somehow activated in opposite environmental conditions. Beclin 1 containing complexes may regulate autophagy during starvation while Beclin 1-independent complexes may up-regulate mTOR during amino acid stimulation. Thus, Vps34/Vps15 can mediate sequential, complementary or opposite responses to nutrient availability depending on the partners and environmental conditions. Clearly, the physiological outcomes of varying Vps34/Vps15 activity are difficult to predict.

Muscle tissue is one of the most adaptable tissues in the body as it needs to respond promptly to different physiological conditions, such as exercise, loading and diet modification. For these reasons skeletal muscle requires a rapid and efficient system for removal of altered organelles, elimination of protein aggregates and disposal of toxic products, which can block proper contraction of sarcomeres. Not surprisingly muscle tissue is one of the most responsive tissue to autophagy activation (Mizushima et al, 2004). Consistently, alterations of autophagosome and lysosome functions have important consequences on skeletal muscle pathophysiology (Sandri, 2010). Both, an excess and a reduced levels of autophagy are detrimental for muscle function. Deficiency in mouse skeletal muscles of *Atg7*, an autophagy gene encoding the E1-like enzyme of ubiquitin-like conjugation systems essential for the autophagosome biogenesis, leads to a 20% reduction of muscle mass and specific force with signs of degeneration that are especially evident in muscle wasting conditions (Masiero et al, 2009). Human myopathies due to lack of collagen VI, such as Ullrich dystrophies and Bethlem myopathies, display defective basal autophagy and symptoms are ameliorated by inducing autophagy with diet or pharmacological interventions (Grumati et al, 2010). Conversely, the accumulation of autophagosomes is a pathognomonic feature in other classes of myopathies including autophagic vacuolar myopathies (AVMs), lysosomal storage diseases, inclusion body myopathy with rimmed vacuoles Paget disease of bone-frontotemporal dementia (IBMPFD) (Nishino, 2003). Interestingly, loss of function mutations in myotubularins, the lipid phosphatases that specifically dephosphorylate PI3P and PI(3,5)P_2_ at the D3 position, have been associated with myopathies and defects in the inhibition of autophagy. In particular, myotubularin 1 (*MTM1*) mutations lead to X-linked myotubular myopathy (Beggs et al, 2010), and Jumpy (*MTMR14*) mutations cause congenital disease centronuclear myopathy (Vergne and Deretic, 2010). However, the involvement of Vps34/Vps15 complexes in these muscle diseases has not been addressed.

Here we investigate the effects of Vps15/Vps34 inactivation in the mouse whole body, in mouse embryonic fibroblasts (MEFs), myotubes and in skeletal muscles after genetic deletion of the *Vps15* gene (also known as *Phosphoinositide-3-Kinase, regulatory subunit 4—PIK3R4*). We show that the deletion of the N-terminus region of Vps15 containing putative catalytic domain is sufficient to affect Vps34 expression and activity. Surprisingly, mutant cells do not show impaired mTOR activation by nutrients, despite a severe effect on viability. The capacity of autolysosome clearance is profoundly impaired in *Vps15*-deficient MEFs, myotubes and muscles *in vivo*, while the induction of autophagy is not blocked. Moreover, *Vps15*^−/−^ muscles suffer of AVM characterized by profound muscle weakness and massive accumulation of autophagosomal and lysosomal structures. Finally, human cells from AVM patients display reduced signs of lysosomal storage disease upon ectopic overexpression of Vps15 and Vps34 complex.

## RESULTS

### Generation of *Vps15*-null mice and cells

**Figure 1:**
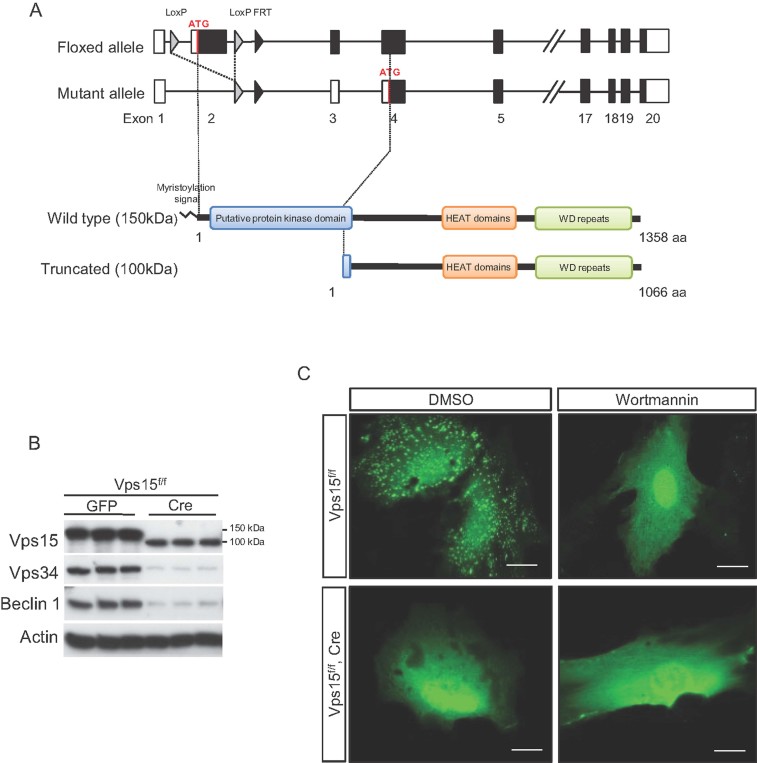
Generation and characterization of *Vps15*-null mice and cells Schematic representation of the targeted allele of *Vps15* gene and Vps15 protein domain structure. The coding exons are depicted by black boxes. The grey triangles denote *loxP* sequences, black triangle—FRT sequence. The putative protein kinase domain, HEAT domains (Huntingtin, elongation factor 3 (EF3), protein phosphatase 2A (PP2A) and the yeast kinase TOR1) and repeats of WD domain in the full-length and truncated Vps15 are depicted.Immunoblot analysis of Vps15, Vps34 and Beclin 1 expression in MEF cells transduced with Adeno-Cre virus analysed 72 h post-infection.*Vps15*-depleted MEFs were transfected with 2xFYVE-GFP expressing plasmid. 36 h post-transfection cells were treated with 200 nM wortmannin or DMSO alone for 30 min and PI3P positive compartments were visualised by confocal microscopy. Scale bars: 20 µm.

To study the function of Vps15 *in vivo* we introduced by homologous recombination two loxP sites flanking exon 2 of the *Vps15* gene. Exon 2 contains the start ATG codon, and encodes the myristoylation signal sequence for membrane localization and putative protein kinase domain including ATP-binding site (Fig. 1A). Vps15 flox/flox (Vps15^f/f^) mice were viable and fertile and did not present any overt phenotype, indicating that the insertion of loxP does not alter *Vps15* gene function. Next, mice were crossed with transgenic mice overexpressing Cre under the human cytomegalovirus minimal promoter (CMV-Cre) (Schwenk et al, 1995) to excise DNA between the loxP sites in all tissues and generate whole body *Vps15*-deficient mice (Vps15^−/−^) (Supporting Information Fig. 1A). Among 250 pups from the intercross of Vps15^f/−^ mice, 168 had the Vps15^f/−^ genotype, 82 were Vps15^f/f^, but no Vps15^−/−^ offspring was found, indicating that *Vps15*-mutant mice die during embryonic development. After timed matings, we did not witness *Vps15*-mutant embryos at as early as embryonic day (E) 7.5. Thus, we conclude that Vps15^−/−^ embryos die during the implantation period before E7.5. The early embryonic lethality is similar to the phenotype of Vps34^−/−^ mice (Zhou et al, 2011), while deletion of the *Atg5* and *Atg7* genes essential for autophagy leads to perinatal lethality (Komatsu et al, 2005; Kuma et al, 2004). Taken together these data are consistent with Vps15 being required for Class III PI3K activity and having a broader role than autophagy regulation (Vanhaesebroeck et al, 2010).

To dissect signalling of *Vps15*-mutant cells, *Vps15* was deleted in cultures of Vps15^f/f^ MEFs by adenoviral transduction of Cre recombinase. As shown in Fig. 1B, Cre expression resulted in the disappearance of full-length Vps15 protein. This was accompanied by the concomitant expression of a truncated Vps15 form of 100 kDa. We hypothesized that this short form arose from translation initiation at a start codon in exon 4 that is in frame with the one in exon 2. RTqPCR experiments confirmed that this short Vps15 version lacked the N-terminus myristoylation signal and putative kinase domain (Supporting Information Fig. 1B). Importantly, the expression of the Vps15 partners, Vps34 and Beclin 1, was decreased after adenoviral Cre transduction (Fig. 1B), consistent with previous data indicating a role for Vps15 in stability of the complex (Thoresen et al, 2010), (Willinger and Flavell, 2012).

Since the best characterized biochemical function of Vps15/Vps34 complex is the production of PI3P (Vanhaesebroeck et al, 2010), we checked whether the PI3P production in *Vps15*-depleted cells was affected. For this purpose we used a reporter construct containing two FYVE domains fused with GFP (2xFYVE-GFP). As FYVE domain binds PI3P in the cell, the fusion to GFP protein gives the opportunity to localize the endogenously produced PI3P (Stenmark and Aasland, 1999). As expected, transiently overexpressed 2xFYVE-GFP protein was localized in vesicular structures in Vps15^f/f^ cells (Fig. 1C), consistent with the reported accumulation of PI3P in endosomes (Gaullier et al, 2000). Treatment with the PI3K inhibitor wortmannin resulted in a diffuse signal of 2xFYVE-GFP protein in Vps15^f/f^ cells, providing a qualitative read-out of PI3P levels in the cell. Importantly, *Vps15* deletion mimicked the effect of wortmannin.

### Induction of autophagosome formation is not impaired in *Vps15*-null MEFs

**Figure 2:**
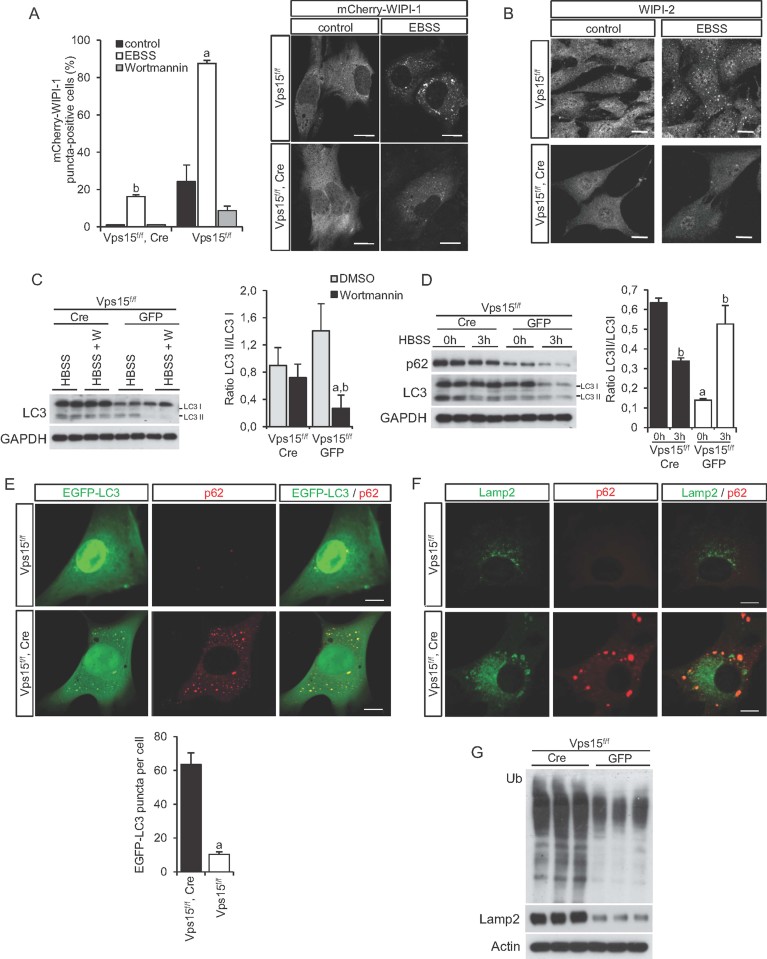
Impaired autophagy in *Vps15*-depleted cells mCherry-WIPI-1 fails to form puncta in *Vps15*-depleted cells. MEFs were treated in nutrient free medium (EBSS) with or without 200 nM wortmannin, for 3 h and images were acquired by confocal microscopy. The number of mCherry-WIPI-1 puncta-positive cells in every treatment condition was determined from 200 individual cells (*n* = 4) by fluorescent microscopy (right panel). Scale bars: 20 µm. Data are mean ± SEM (*p* ≤ 0.01 a: *vs.* control b: *vs.* Vps15^f/f^) (left panel).Endogenous WIPI-2 fails to form puncta in *Vps15*-depleted cells. MEFs were starved in nutrient free medium (EBSS) for 3 h and then fixed. WIPI-2 positive structures were visualized by immunofluorescence using anti-WIPI-2 antibody and images were acquired by confocal microscopy. Scale bars: 20 µm.Immunoblot analysis of unprocessed and lipidated form of LC3 protein in *Vps15*-depleted cells. Cells were starved in HBSS medium with or without 200 nM wortmannin for 1 h. For protein analysis, the ratio of the densitometric assay of the two LC3 forms is presented. Data are mean ± SEM (*p* ≤ 0.05 a: *vs.* CRE; b: *vs.* DMSO).Immunoblot analysis of p62 and LC3 proteins in *Vps15*-depleted cells. To induce autophagy cells were starved in HBSS medium for 3 h. The ratio of the densitometric assay of the two LC3 forms is presented. Data are mean ± SEM (*p* ≤ 0.05 a: *vs.* CRE; b: *vs.* 0 h).p62-positive structures are co-localized with LC3 in *Vps15*-depleted MEFs. *Vps15*-depleted MEFs were infected with EGFP-LC3 expressing adenoviral vector and analysed by immunofluorescence using anti-p62 antibody. EGFP-LC3 puncta were counted in cells (*n* = 40 in Vps15^f/f^, Cre and *n* = 25 in Vps15^f/f^. Data are mean ± SEM (*p* ≤ 0.05 a: *vs.* CRE).Double-immunofluorescence staining of control and *Vps15*-depleted MEFs using anti-p62 and anti-LAMP2 antibodies. Scale bars: 10 µm.Immunoblot analysis of total protein extracts of *Vps15*-depleted and control MEFs cells using anti-ubiquitin and anti-LAMP2 antibodies.

Autophagy is a major process controlled by the Vps15/Vps34 complex in yeast and mammalian cells (Simonsen and Tooze, 2009), though the level of regulation and the *in vivo* implications are unclear and require further work. Major PI3P-binding effectors that facilitate the formation of autophagosomes are the WD repeat domain phosphoinositide-interacting proteins 1 and 2 (WIPI-1 and 2), mammalian orthologues of yeast Atg18 (Codogno et al, 2011). Therefore, we transiently overexpressed mCherry tagged WIPI-1 protein in control Vps15^f/f^ MEFs and after *Vps15* deletion by adenoviral Cre transduction. As expected, starvation in nutrient-deprived medium caused a sharp increase in mCherry-WIPI-1 positive puncta that was sensitive to wortmannin treatment in control cells (Fig. 2A). However, the number of cells presenting with mCherry-WIPI-1 positive puncta after starvation was dramatically lower in *Vps15*-depleted cells, although not completely abrogated. In addition, the size of the puncta was also reduced in mutant cells. These results were confirmed by immunostaining with antibodies directed against endogenous WIPI-2 (Fig. 2B). Taken together, our findings with FYVE-domain containing reporter proteins and endogenous PI3P effectors indicate that PI3P levels are sharply reduced upon *Vps15* depletion and that alternative mechanisms for PI3P production and WIPI regulation through other classes of PI3K or PIP phosphatases do not play a major compensatory role in *Vps15*-deficient MEFs.

Having demonstrated an impairment of PI3P signal transduction in *Vps15*-deficient cells, we asked the functional consequences on the autophagosome formation. The appearance of a faster migrating band of LC3 protein due to its lipidation and cleavage is a common marker of autophagy induction (Klionsky, 2007). As expected, in control Vps15^f/f^ cells LC3 lipidation was blocked by wortmannin (Fig. 2C), consistent with a role of PI3K in autophagy initiation. However, in Cre-transduced cells, LC3 levels of both unlipidated and lipidated forms were increased as compared to GFP-transduced control, and lipidation was largely insensitive to wortmannin treatment. Strikingly, the increased levels of unlipidated and lipidated LC3 in Cre-transduced cells were also detected in basal nutrient-rich conditions (Fig. 2D). Next, we measured the levels of p62 protein, that functions as a cargo receptor for degradation of ubiquitinated proteins by the autophagosome (Rusten and Stenmark, 2010). Similar to LC3, p62 levels accumulated in *Vps15*-deficient cells even in basal conditions (Fig. 2D). To visualize autophagy, we performed microscopic analyses of *Vps15*-depleted and control cells to observe the presence of LC3 and p62 positive structures by EGFP-LC3 overexpression and immunostaining with anti-p62 antibody. In control cells in the presence of nutrients and growth factors the EGFP-LC3 staining was diffuse and p62 positive vesicles were scattered, indicating a low rate of autophagy (Fig. 2E). Strikingly, in *Vps15*-deficient cells numerous EGFP-LC3 and p62 positive structures were evident in nutrient-rich conditions. Counts of LC3 puncta showed a sixfold increase in mutant cells as compared to control (Fig. 2E). A significant fraction of p62 positive structures were also labelled by co-immunostaining with anti-Lamp2 or anti-Lamp1 antibodies, two common lysosomal markers (Fig. 2F and Supporting Information Fig. 2). Concomitantly, a sharp increase of ubiquitinated proteins and Lamp2 levels was detected in mutant cells using immunoblot analysis (Fig. 2G). In conclusion, LC3-positive autophagosomes are detected in *Vps15*-deficient cells, even in nutrient-rich conditions. Since LC3 and p62 are also substrates of the autophagy pathway, the massive amount of lysosomes containing p62 suggests impairment in terminal stages of autophagy after the fusion with lysosomes, leading to an accumulation of ubiquitinated proteins. Our findings suggest that the major functional defect after *Vps15* deletion in MEFs is subsequent to LC3 processing and autophagosome formation that in mutant cells might be triggered by a wortmannin-insensitive pathway.

### Autophagy flux is compromised in *Vps15*-depleted cells

**Figure 3:**
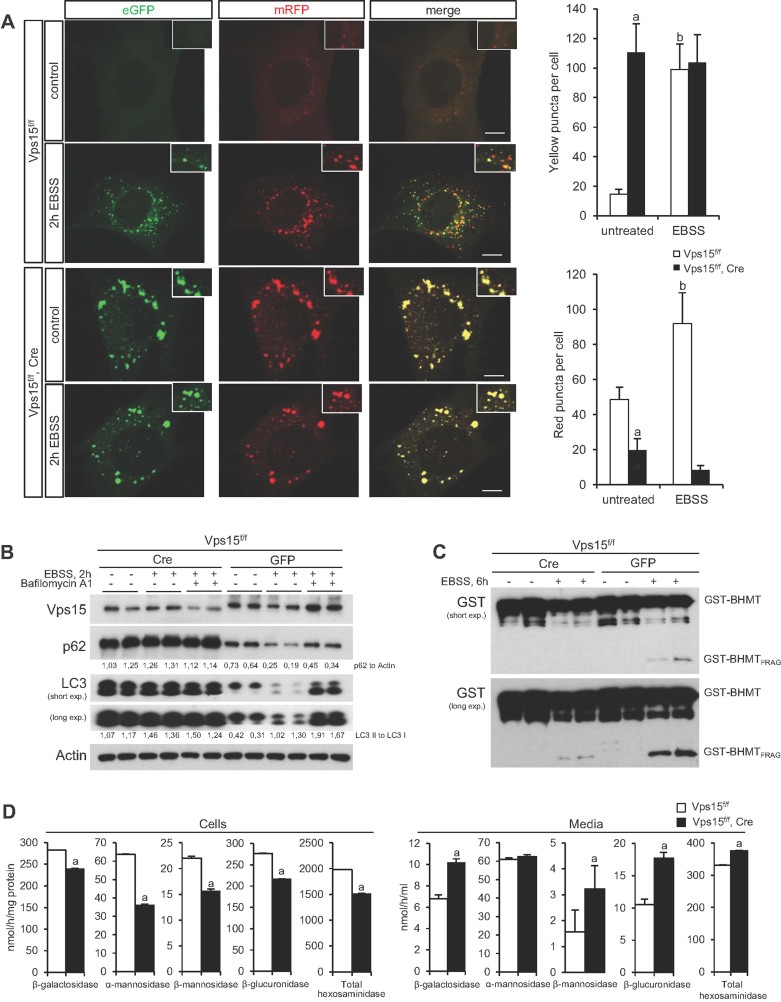
Autophagy flux is diminished in *Vps15*-depleted MEFs *Vps15*-depleted and control MEFs were transduced with tfLC3 adenovirus. 48 h post-infection cells were either kept in full medium or starved in EBSS medium for 2 h, fixed and analysed by microscopy. Insets show higher magnification views. Counts of average number of yellow and red puncta per cell is presented on the graphs. Data are mean ± SEM (*n* = 40, *p* ≤ 0.05, a: *vs.* Vps15^f/f^ cells, b: *vs.* untreated). Scale bars: 10 µm.Immunoblot analysis of total protein extracts of *Vps15*-depleted MEFs treated with EBSS for 2 h with or without 200 nM bafilomycin A1 using indicated antibodies. The ratio of p62 to actin and ratio of two LC3 forms of the densitometric assay is presented.Immunoblot analysis of the Glutation sepharose eluates using anti-GST antibody. *Vps15*-depleted or control MEFs were transfected with plasmid expressing GST-BHMT, 24 h post-transfection cells were treated with EBSS supplemented with non-essential amino acids, E64d (6 µM) and leupeptin (11 µM) for 6 h to induce accumulation of lysosome-derived GST-BHMT proteolytic fragment. Total proteins were extracted and GST-fusion fragments precipitated using Glutation sepharose.Lysosomal enzymes activity in the extracts of control and *Vps15*-depleted MEFs and in culture media. Equal numbers of *Vps15*-depleted or control cells were plated. The media was changed to fresh 24 h before collecting. The cell pellets and culture media were collected at day 6 post-infection. Data are mean ± SEM (*n* = 6, *p* ≤ 0.05 a: *vs.* Vps15^f/f^).

To thoroughly follow autophagy flux from initiation to termination, we used monomeric Red Fluorescent Protein–Enhanced Green Fluorescent Protein (mRFP-EGFP) tandem fluorescent-tagged LC3 (tfLC3) (Kimura et al, 2007). tfLC3 emits both EGFP and mRFP signals before the autophagosome fusion with the lysosomes. However, the acidic lysosomal environment quenches the EGFP signal, while preserving the mRFP signal. In control MEFs after nutrient starvation, the numbers of both yellow (merged EGFP and mRFP signals) and red puncta were significantly increased, indicating active formation of both autophagosomes and autolysosomes (Kimura et al, 2007). Consistent with the analyses shown in Fig. 2, in *Vps15*-depleted cells the yellow puncta representing autophagosomes were predominant and detected both in basal and starved conditions (Fig. 3A). Importantly, the red puncta representing autolysosomes in mutant cells were lower as compared to control and were not induced by starvation. Next, we followed LC3 processing and p62 levels in presence of the lysosomal inhibitor bafilomycin A1, a known specific inhibitor of vacuolar-type H^+^-ATPase (Fig. 3B and Supporting Information Fig. 3A). While in control cells this treatment led to the accumulation of processed LC3, in mutant cells the high levels of processed LC3 detected in basal conditions were not further increased by bafilomycin A1 treatment. In parallel, the p62 levels in mutant cells were also refractory to starvation and bafilomycin A1 treatment.

To further address lysosomal function in *Vps15*-depleted cells we employed betaine homo-cysteine methyltransferase (BHMT) assay (Mercer et al, 2008). In this assay the lysosomal activity is monitored by following the degradation of ectopically overexpressed GST-BHMT fusion protein. Previously, it has been established that GST-BHMT serves as a reporter of the autophagic flux as its delivery to the lysosome and degradation is dependent on macroautophagy pathway (Dennis and Mercer, 2009). As presented in Fig. 3C, upon autophagy induction condition the fragmentation of GST-BHMT reporter is severely impaired in *Vps15*-depleted MEFs compared to control cells. Taken together, our data are consistent with compromised Vps15/Vps34 signalling causing a block in autophagy flux after LC3 processing and autophagosome formation, and before lysosomal clearance. To address the possible cause of the low lysosomal activity in *Vps15*-depleted cells we assayed the activities of several lysosomal enzymes. As presented in Fig. 3D, the activities of the tested lysosomal enzymes were significantly decreased in the extracts of *Vps15*-depleted cells as compared to control cells. Concurrently, we detected a significant increase in the activities of the same enzymes with the exception of α-mannosidase in the cell culture media. These results indicate that lysosomal enzymes are mistargeted in *Vps15*-depleted cells during the endosomal transport from the trans Golgi network and are excreted to the culture medium, contributing to the low lysosomal activity observed in mutant cells.

### Nutrient signalling to mTOR is not significantly affected in *Vps15*-null MEFs

**Figure 4:**
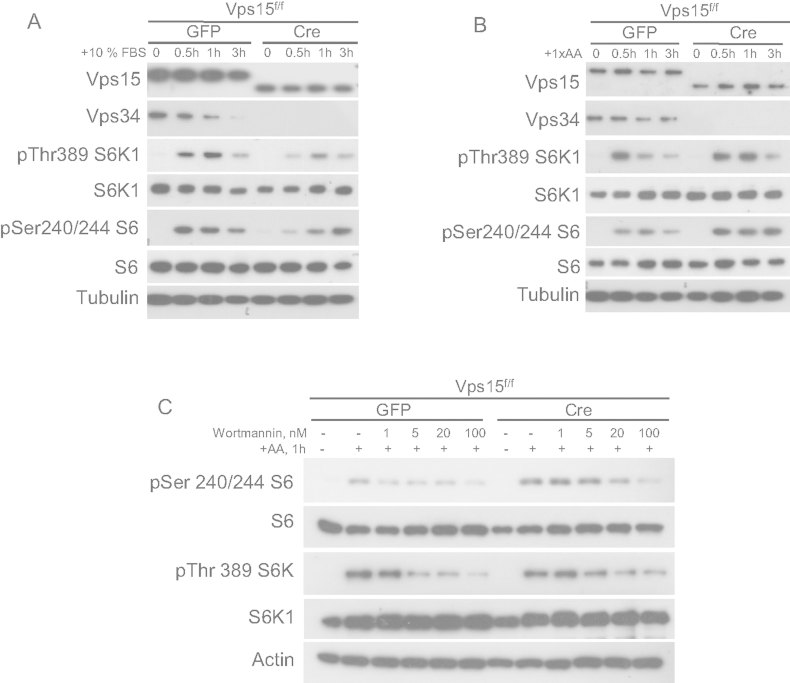
Growth factor and nutrient signalling in *Vps15*-depleted MEFs **A,B.** Growth factor and nutrient stimulated signalling in *Vps15*-depleted cells. GFP or CRE adenovirus transduced MEFs were nutrient starved and then stimulated with dialysed FBS or cocktail of amino acids for indicated times. Total protein extracts were immunobloted with indicated antibodies.**C.** Dose-dependent inhibition of mTOR activation by wortmannin. Immunoblot analysis with indicated antibodies of GFP or CRE adenovirus transduced MEFs which were nutrient starved and then treated with indicated doses of wortmannin or DMSO for 1 h before stimulation with a cocktail of amino acids for additional 1 h.

Whether the Vps15/Vps34 complex directly regulates amino-acid sensing and mTOR complex 1 (mTORC1) activation, is still an open question with conflicting reports (Juhasz et al, 2008; Nobukuni et al, 2005; Yoon et al, 2011; Zhou et al, 2011). In addition, the alteration of lysosomal mass and p62 levels that we observe in *Vps15*^−/−^ cells, could also indirectly impact on mTORC1 activation by amino-acids, as lysosomes and p62 play an important role in this regulatory mechanism (Duran et al, 2011; Zoncu et al, 2011). To address the status of mTOR signalling in *Vps15*-depleted cells, we treated nutrient starved cells with growth factors in dialysed serum or with amino acid mixture, and assessed mTORC1 activity by immunoblot analysis with antibodies against phospho-Thr389 S6K1 or phospho-Ser240/244 rpS6. As shown in Fig. 4A, *Vps15* depletion led to a minor decrease and slower kinetics of mTORC1 activation by growth factors. Upon amino acid stimulation phosphorylation of mTORC1 targets was not dramatically changed even if we observed a tendency of mTORC1 activity to be increased over prolonged time period in cells lacking Vps15 (Fig. 4B). Our data do not support a positive requirement for Vps15/Vps34 in amino acid-stimulated mTOR activity. The minor activation of mTORC1 signalling by amino acids observed in *Vps15*-deficient cells may be consistent with the adaptive augmentation in lysosome mass and p62 levels observed in these cells (Fig. 2F). Interestingly, the PI3K inhibitor wortmannin decreased amino acid stimulated mTOR-signalling in both wild type and *Vps15*-deficient cells with similar potency (Fig. 4C), suggesting that additional PI3K classes distinct from Class III participate in this regulation.

To rule out the potential contribution in observed phenotype of the truncated Vps15 form that was generated by our gene targeting strategy, we overexpressed the 100 kDa Vps15 protein by adenoviral transduction in wild type cells. As shown in the Supporting Information Fig. 3B, the truncated Vps15 did not affect the lysosomal mass as measured by Lamp2 immunoblot, the p62 and LC3 levels, the LC3 lipidation as well as the mTORC1 signalling. Furthermore, unlike full-length endogenous Vps15, the truncated 100 kDa Vps15 protein could not be immunoprecipitated in the complex with Beclin 1 or Vps34 (Supporting Information Fig. 3C). Thus the truncated Vps15 cannot form protein complexes with Class III PI3K activity and does not acquire signalling properties that could explain the effects of the gene targeting strategy on lysosomal function and mTOR signalling.

### Deletion of *Vps15* in skeletal muscles results in severe muscle damage

**Figure 5:**
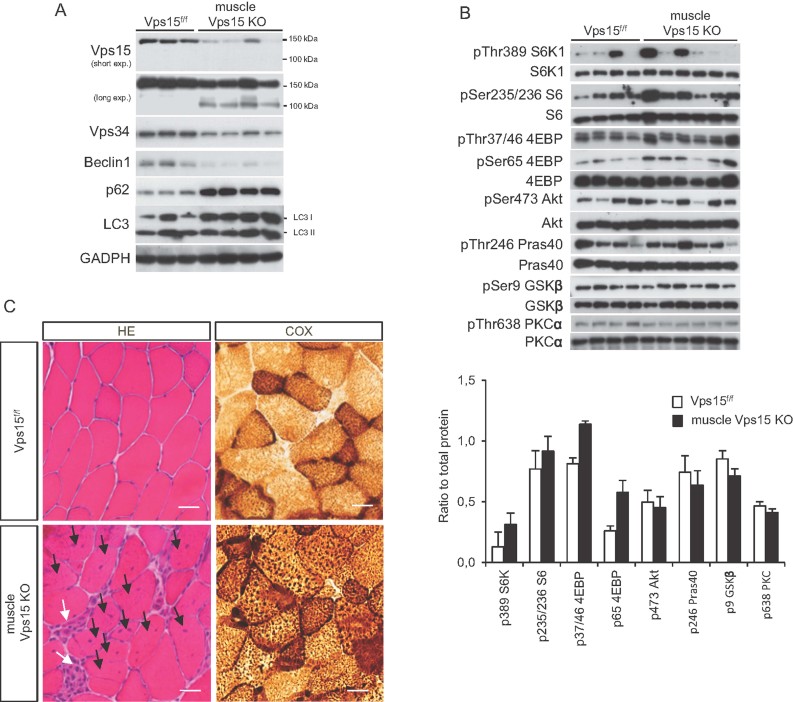
Generation and characterization of muscle-specific*Vps15*-knockout mice Immunoblot analysis of TA muscle of 2 month old muscle Vps15 KO and matching control mice with indicated antibodies.mTORC1 and mTORC2 activity in TA muscles of 2 month old muscle Vps15 KO and matching control mice analysed by immunoblotting with indicated antibodies. The ratio of phosphorylated to total protein of the densitometric assay is presented. Data are mean ± SEM.Histological analysis of muscles by HE staining and COX activity from 2 month old muscle Vps15 KO and matching control mice. Black arrows indicate centronucleated fibres; white arrows—cell infiltration. Scale bars: 40 µm.

To address the physiological function of Vps15 *in vivo* and to overcome the embryonic lethality of *Vps15*^−/−^ mice, we generated skeletal muscle specific knockout of *Vps15* (muscle Vps15 KO), by crossing *Vps15*^*f/*f^ mice with a transgenic line expressing Cre recombinase under the control of Human Skeletal Actin promoter (Miniou et al, 1999). The presence of deleted allele was confirmed by PCR analysis of genomic DNA purified from skeletal muscle (Supporting Information Fig. 4A). Muscle Vps15 KO mice were born at the expected Mendelian ratio and had similar growth curves as compared to Cre-negative littermates (Supporting Information Fig. 4B). In addition, muscle Vps15 KO mice did not present an overt deterioration in nutrient homeostasis, as indicated by the normal glucose tolerance test (GTT) and insulin tolerance test (ITT) (Supporting Information Fig. 4C and D). Depletion of Vps15 was confirmed by immunoblot analyses of tibialis anterior (TA) muscle from 2 month old animals where Vps15 protein levels were significantly reduced (Fig. 5A). The residual detection of full length Vps15 protein in the muscle extracts from mutant mice may be due to incomplete excision, though the contribution of cell types other than differentiated muscle fibres within the tissue could not be excluded. Depletion of *Vps15* in skeletal muscles resulted in decreased levels of Vps34 and Beclin 1 partners in the complex, and accumulation of p62 and LC3. Consistent with the observations in amino acid-stimulated MEFs, a minor activation of mTORC1 signalling to S6K1 and 4E binding protein 1 (4EBP) was observed in Vps15 KO muscles from randomly fed and starved/refed animals (Fig. 5B and Supporting Information Fig. 5). Morphometric examinations did not reveal significant changes in fibre type composition and cross section area in Vps15 KO muscles (Supporting Information Fig. 6A and B). However, severe degenerative changes were evident in the *Vps15*-depleted muscles including appearance of necrotic fibres, cell infiltration, centronucleated fibres as early as 2 months after birth in all types of muscles examined (Fig. 5C, Supporting Information Fig. 6C). The infiltration detected in the *Vps15*-depleted muscles consisted of immune cells, macrophages and T-cells, as revealed by immunostaining with the F4/80 and CD3 markers, respectively (Supporting Information Fig. 7A). Furthermore, the induction of the inflammatory response in *Vps15*-depleted muscles was confirmed at the transcript level by the detection of inflammatory cytokines and cell markers (Supporting Information Fig. 7B). The morphological changes observed in *Vps15*-depleted muscles were accompanied by marked changes in mitochondrial content as assessed by cytochrome c oxidase (COX) assay (Fig. 5C).

### Muscle *Vps15* knockout results in autophagy block distinct from *Atg7* knockout

**Figure 6:**
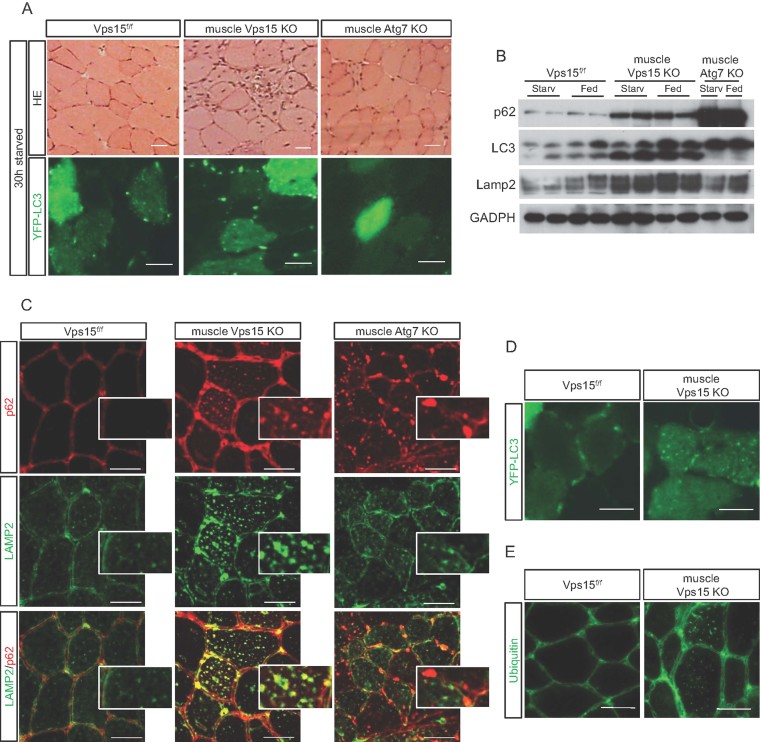
Muscle-specific deletion of *Vps15* is distinct from muscle-specific *Atg7* knockout Histological analysis of TA muscles of 4 month old 30 h starved muscle Atg7 KO mice, muscle Vps15 KO mice and matching control mice by HE staining and YFP-LC3 fluorescence. Scale bars: 20 µm (HE) and 40 µm (YFP-LC3).Immunoblot analysis of total protein extracts of gastrocnemius muscles with indicated antibodies. Gastrocnemius muscles were dissected from 30 h starved and random fed 4 month old muscle Atg7 KO mice, muscle Vps15 KO mice and matching control mice.Accumulation and co-localization of p62/LAMP2 positive structures in TA muscle of 4 month old random fed muscle Vps15 KO, muscle Atg7 KO and matching control mice. Insets show higher magnification views. Scale bars: 40 µm.Histochemical analysis of TA muscle of randomly fed 4 month old muscle Vps15 KO and matching control mice by LC3-YFP fluorescence. Scale bars: 40 µm.Immunostaining of TA muscle of randomly fed 4 month old Vps15 muscle KO and matching control mice using anti-ubiquitin antibody. Scale bars: 40 µm.

Genetic mouse models carrying muscle specific inactivation of autophagy genes, *Atg7* (Masiero et al, 2009) and *Atg5* (Raben et al, 2008), highlighted the role of macroautophagy in the maintenance of muscle mass and function. We took advantage of already generated muscle-specific *Atg7* knockout mice (muscle Atg7 KO) and set up a comparative analysis of phenotypes with Vps15 muscle KO (Masiero et al, 2009). For quantitative comparative analysis of *Vps15* and *Atg7* mutants we mainly focused on TA muscles that are predominantly composed by fast twitch fibres and thus relatively homogenous in terms of fibre typing. As shown in Fig. 6A, at the age of 4 months the severity of muscle damage was more pronounced in fast twitch TA muscles from Vps15 KO mice compared to Atg7 KO, as judged by the number of necrotic fibres, centronucleated fibres and cell infiltration. These data point to a broader role of *Vps15* gene in muscle function than the autophagy genes.

After electroporation of TA muscles with YFP-LC3 overexpressing plasmid and after 30 h starvation, Atg7 KO muscles failed to form YFP-LC3 positive autophagosomes, as opposed to starved control mice and muscle Vps15 KO mice (Fig. 6A). Consistently, LC3 was not processed in Atg7 KO mice, whereas Vps15 KO mice displayed an up-regulation of both total and lipidated LC3 as compared to control (Fig. 6B). However, p62 accumulated in both mutants, with higher levels observed in *Atg7*-deficient muscles (Fig. 6B and C). Interestingly, p62 protein appeared differentially localized in Atg7 and Vps15 mutants. While in *Vps15*-deficient TA muscles p62 positive structures were uniformly distributed and largely co-labelled with Lamp2 antibodies representing autolysosomes, in Atg7 mutants p62 was detected in subsarcolemmal protein aggregates void of Lamp2 staining (Fig. 6C). The selective increase in the amount of lysosomes observed in *Vps15*-depleted muscles (Fig. 6C) was confirmed by immunoblot analysis (Fig. 6B) and was unlikely to be due to an induction of lysosomal biogenesis, the expression of the master regulator of lysosome biogenesis TFEB and its downstream targets were unaffected at the transcript level (Supporting Information Fig. 8). In contrast to the control mice, p62 and YFP-LC3 positive structures were evident in muscle Vps15 KO mice that were *ad libitum* fed and did not undergo a starvation protocol (Fig. 6C and D). In addition, *Vps15*-deficient fibres contained high levels of ubiquitinated proteins, as detected by immunostaining (Fig. 6E). To evaluate possible phenotypic differences at early times after gene deletion, we established primary myoblast cultures from Vps15^f/f^ mice, differentiated the myoblasts to myotubes *in vitro* and transduced them with adenoviral Cre to induce gene deletion. In this cell autonomous model we observed that depletion of *Vp15* results in a phenotype similar to the one observed in MEFs as judged by the induction of p62, LC3 and the sparing of mTOR signalling (Supporting Information Fig. 9A and B). Furthermore, by directly comparing *Vps15* and *Atg7* deletion in the myotube cultures we observed that depletion of both genes led to marked accumulation of p62 (Supporting Information Fig. 9C). However, EGFP-LC3 positive puncta were undetectable in *Atg7*-depleted cells even in nutrient-deprived medium, while they were constitutively present in *Vps15*-depleted cells even in nutrient-rich conditions (Supporting Information Fig. 9D). These observations recapitulated data of our *in vivo* Atg7 and Vps15 KO muscles analysis. In conclusion, defects in p62 and ubiquitinated protein degradation in *Vps15*-deficient muscles are accompanied by an up-regulation of LC3 positive autophagosomes and Lamp2 positive lysosomes, which is not observed in *Atg7*-deficient muscles.

### Ultrastructural analyses of Vps15 KO muscles

**Figure 7:**
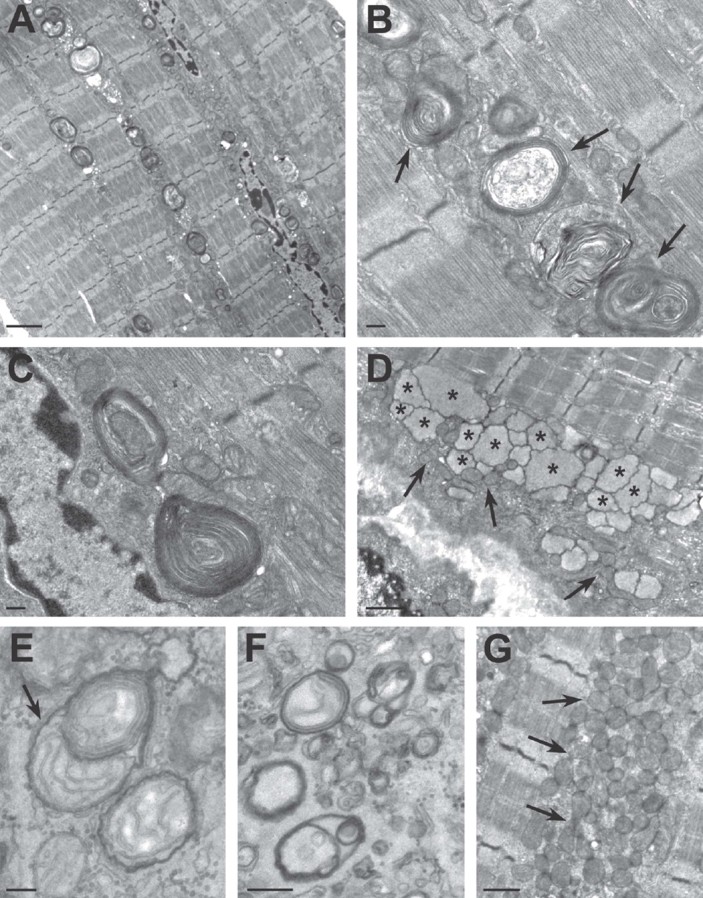
Ultrastructural analysis of *Vps15*-deficient muscles Electron micrographs of *Vps15*-deficient EDL muscles. Low magnification micrograph showing accumulation along the fibre of numerous autophagic vacuoles of varying morphologies.Enlargement of autophagic vacuoles and vacuoles with lamellated membrane structure (black arrow).Enlargement of a large multilamellar structure in proximity to the nucleus.Accumulation of mitochondria (black arrows) and lysosome like structures (black asterisks).Mitochondria enclosed in an autophagic vacuole (black arrow).Autophagic vacuoles at different stages.Accumulation of mitochondria (black arrows). Scale bars: A and E, 2 µm; B, C and F, 0.2 µm; D and G, 1 µm.

To unquestionably identify the morphological alterations of *Vps15*-deficient tissue, EDL muscles underwent electron microscopy (EM) analysis. As shown in Fig. 7A, *Vps15*-deficient muscles displayed massive accumulation of vacuoles that were frequently found in longitudinal stacks. At higher magnification a large fraction of these vacuoles appeared constituted by variable number of membrane layers (Fig. 7B and C). The various appearance of other vacuoles represented different stages of maturation (*e.g*. lysosomes in Fig. 7D, autophagosome in Fig. 7E). Importantly, autophagosomes, constituted by two layers of membranes enclosing degraded material or organelles (*i.e*. a mitochondrion in Fig. 7E) were frequently observed (Fig. 7E and F). Furthermore, abnormally shaped mitochondria were accumulated and active mitochondria autophagy (mitophagy) was observed (Fig. 7E and G). Thus, EM analysis provided the demonstration that autophagosomes and lysosomes accumulate in *Vps15*-deficient muscles.

### Muscle Vps15 KO mice display symptoms of autophagic vacuolar myopathies

**Figure 8:**
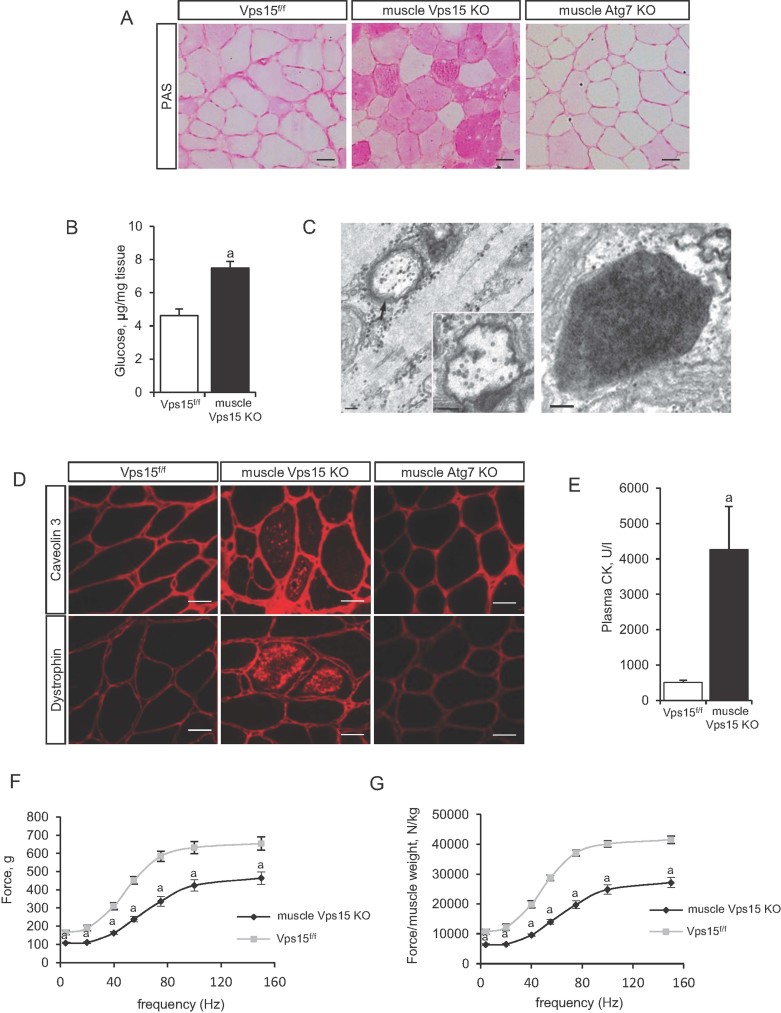
Muscle-specific deletion of *Vps15* manifests with signs of lysosomal storage disease Deletion of *Vps15* affects glycogen metabolism in muscles. Histological analysis of TA muscles of 4 month old starved muscle Atg7 KO, muscle Vps15 KO and matching control mice by PAS staining to detect glycogen. Scale bars: 40 µm.Glycogen was extracted and levels determined by enzymatic assay in TA muscles of 4 month old randomly fed muscle Vps15 KO and matching control mice. Data are mean ± SEM (*p* ≤ 0.05 a: *vs.* Vps15^f/f^).Electron micrographs of *Vps15*-deficient EDL muscle fibre showing accumulation of glycogen granules (black dots) in the cytoplasm, inside the double membrane structures (left) and in lysosome (right). Scale bar: 0.2 µm.Immunostaining of TA muscle of randomly fed 4 month old muscle Atg7 KO mice, muscle Vps15 KO mice and matching control mice using anti-Dystrophin and anti-Caveolin 3 antibodies. Scale bars: 40 µm.Plasma creatine kinase levels of randomly fed 4 month old muscle Vps15 KO and matching control mice. Data are mean ± SEM (*n* = 4–8, *p* ≤ 0.05 a: *vs.* Vps15^f/f^).*In vivo* force measurements performed on gastrocnemius muscle of 4 month old muscle Vps15 KO and matching control mice during tetanic contraction. Data are mean ± SEM (*n* = 5 *p* ≤ 0.05 a: *vs.* Vps15^f/f^).Relative force measurements after normalization of absolute tetanic force to the gastrocnemius muscle weight. Data are mean ± SEM (*n* = 5 *p* ≤ 0.05 a: *vs.* Vps15^f/f^).

Accumulation of autophagosomes and muscle damage in Vps15 KO mice were reminiscent of AVMs (Nishino, 2003). These are rare genetic diseases characterized by abnormal lysosomal function. The best characterized AVM is Danon disease, in which the causative gene is *Lamp2* (Nishino et al, 2000). Similarly to *Vps15*-depleted MEFs and muscles, Lamp2 mutant cells are characterized by autophagic build-up, intracellular mistargeting of lysosomal enzymes which is accompanied by increased lysosomal enzyme secretion (Eskelinen et al, 2002). We, therefore, addressed whether muscle Vps15 KO mice displayed distinctive features of AVMs, namely glycogen accumulation and autophagic vacuoles with sarcolemmal features. Analysis of glycogen levels by Periodic acid Schiff (PAS) staining revealed a sharp increase in glycogen accumulation in *Vps15*-deficient muscles as compared to control and *Atg7*-deficient muscles (Fig. 8A). Biochemical analysis confirmed a 60% increase in glycogen levels in Vps15 mutants (Fig. 8B). Using an embedding procedure that allows visualizing glycogen granules as electron-dense small dots, the presence of glycogen was evident in the cytoplasm and inside of both the autophagosomes and the lysosomes by EM (Fig. 8C and Supporting Information Fig. 10). Thus, glycogen is accumulated in Vps15 mutants, distinct to Atg7 mutants.

Presence of the sarcolemmal features is a well-described diagnostic feature of muscles in AVM patients. Proteins which are under normal conditions localized to the membrane (*e.g*. dystrophin, acetylcholinesterase, caveolin 3), are mislocalized and are found inside the affected muscle fibres within the vacuolar structures. Of note, *Vps15*-deficient muscles, but not *Atg7*-deficient muscles, presented with the accumulation of all above-mentioned proteins in the vacuoles within the fibres (Fig. 8D and Supporting Information Fig. 11A). Furthermore, the phenotype of Vps15 muscle KO mice was highly reminiscent of the phenotype observed in muscles of *Lamp2* knockout mice (model of Danon disease) which presented a similar accumulation of glycogen and p62, as well as sarcolemmal features (Supporting Information Fig. 11B). In line with the severe muscle damage in Vps15 muscle KO mice, the levels of plasma creatine kinase were increased by eightfold, another commonly observed symptom in AVM patients (Fig. 8E). To assess the functional consequences of *Vps15* deletion, *in vivo* force measurements on the gastrocnemius muscles were performed in 4 month old mice through stimulation of the sciatic nerve. These analyses showed a significant reduction in absolute force (Fig. 8F), and relative force after normalization to muscle mass (Fig. 8G). Quantitative decrease in muscle force in Vps15 mutants was more prominent as compared to reported in Atg7 mutants (Masiero et al, 2009), and was close to dystrophic mdx mice (Head et al, 1992), indicating a general reduction in force generation and profound muscle weakness.

### Class III PI3K overexpression alleviates glycogen accumulation in human AVM muscle cells

**Figure 9:**
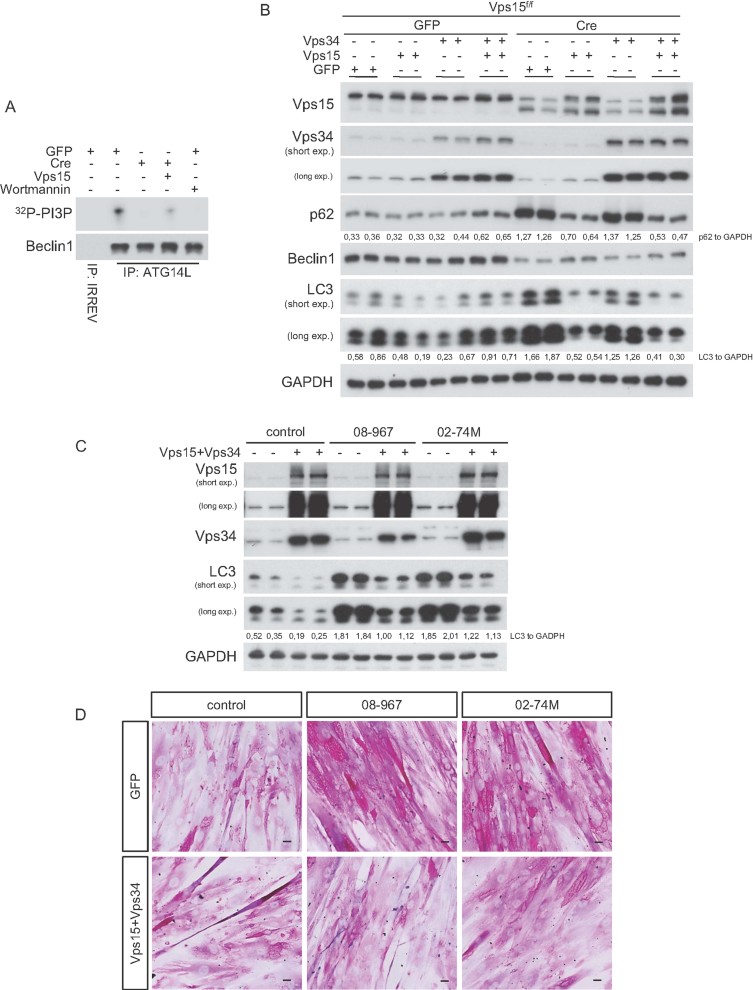
Overexpression of Vps15/Vps34 complex rescues autophagy defects in *Vps15*-depleted MEFs and upregulates autophagy in primary myotube cultures from Danon disease patients improving the glycogen metabolism Intact Vps15 is required for Vps34 kinase activity. Beclin 1 complexes were immuno-purified with antibody against ATG14L from *Vps15*-depleted MEFs, *Vps15*-depleted MEFs transduced with indicated adenoviral vectors or from control MEFs in which Vps34 was pharmacologicaly inactivated. Activity of Vps34 complexes was assayed by an *in vitro* kinase assay with phosphatidylinositol and ^32^P-ATP as substrates. ^32^P-PI3P was resolved by thin layer chromatography and visualized by autoradiography. Fractions of the kinase reaction were used for immunoblotting for Beclin 1.*Vps15*-depleted MEFs were transduced with indicated adenoviral vectors, cells collected 60 h post-infection and total protein extracts immunobloted with indicated antibodies. The ratio of p62 or LC3 to GAPDH of the densitometric assay is presented.Human myoblasts derived from Danon disease patients were differentiated to myotubes and then transduced 2 days post-differentiation with indicated adenoviral vectors. Cells were collected 3 days post-infection and total protein extracts were analysed by immunoblotting with indicated antibodies. The ratio of LC3 to GAPDH of the densitometric assay is presented.PAS staining of the glycogen in human myotubes derived from Danon disease patients with and without Vps15 and Vps34 overexpression, analysed 3 days post-infection. Scale bars: 20 µm.

As a first step to establish gain-of-function approaches, we asked whether adenoviral mediated overexpression of Vps15 and/or Vps34 in *Vps15*-deficient MEFs was sufficient to rescue the autophagolysosome defects. The overexpression of Vps15 was able to restore the levels of PI3P in the *Vps15*-depleted MEFs, as judged by the localization of 2XFYVE-GFP protein and the measurements of PI3P kinase activity in immunocomplex kinase assays after pull down with antibodies against the Vps15 partners Atg14L and Vps34 (Fig. 9A; Supporting Information Fig. 12A and data not shown). Importantly, as shown in the Fig. 9B and Supporting Information Fig. 12B, the overexpression of Vps15 in *Vps15*-depleted MEFs completely reverted the accumulation of p62 and LC3, indicating an efficient autophagic flux. Interestingly, the overexpression of Vps34 in *Vps15*-deficient cells was not sufficient to rescue the autophagy flux and PIP3s levels, confirming that Vps15 not only regulates Vps34 stability but is also required for PI3K activity (Fig. 9B and Supporting Information Fig. 12B). Importantly, overexpression of the truncated 100 kDa Vps15, unlike the full-length protein, did not further worsen the phenotype of Vps15-mutant cells and was not able to rescue neither PI3P levels nor accumulation of Lamp2, p62, LC3 (Supporting Information Fig. 13A–C).

Next, we asked whether these gain-of-function approaches may be beneficial in human muscle cells from AVM patients (Nishino et al, 2000). Danon disease is caused by point loss-of-function mutations in Lamp2 gene leading to lysosomal storage disease. One prominent clinical feature in these patients is the accumulation of glycogen within the muscle fibres (Malicdan et al, 2008). As shown in Fig. 9C, overexpression of both Vps15 and Vps34 in two different cell lines of Danon disease patients can partially decrease the LC3 levels in these cells. Importantly, this effect was concomitant with a reduction in glycogen accumulation as judged by PAS staining (Fig. 9D). Notably, overexpression of Vps15 alone was not sufficient to improve the phenotype of the patient cells, consistent with the data indicating that both Vps15 and Vps34 should be expressed to induce production of PI3P in mammalian cells (Supporting Information Fig. 14A) (Yan et al, 2009). Importantly, overexpression of the truncated 100 kDa Vps15 protein did not affect LC3 or glycogen levels in the patient cells (Supporting Information Fig. 14B and C). Our data are consistent with a critical role of the Vps34/Vps15 complex in regulating autolysosome and endosomal function that are affected in AVMs and other lysosomal storage diseases.

## DISCUSSION

To dissect the complex and controversial role of Vps15/Vps34 in autophagy, nutrient sensing and mTOR signal transduction, we generated *Vps15* loss-of-function mutants in MEFs, myotubes and skeletal muscles. While our findings do not support an essential role for Vps15/Vps34 in autophagy initiation and mTOR activation in these cell types, we uncover severe defects in autophagy maturation, lysosomal function, glycogen storage that in skeletal muscles cause AVM with sarcolemmal features, muscle weakness and lysosomal storage disease.

Our data are consistent with a complete loss-of-function of Class III PI3K activity in *Vps15*-mutant cells. Vps15 mutant mice die during early embryonic development before E7.5, similar to the *Vps34* knockout embryos (Zhou et al, 2011). In addition, Cre expression in MEFs leads to a sharp decrease in Vps34 levels few days after *Vps15* excision. This effect is paralleled by a drop in endosomal 2xFYVE-GFP and PI3K kinase activity in Atg14L immunoprecipitates, that is comparable in wortmannin-treated cells and can be rescued by adenoviral transduction of full length Vps15 (Fig. 1C; Fig. 9A and Supporting Information Fig. 12A and 13B). Since the truncated Vps15 protein that is detected after gene targeting, does not form a complex with Vps34 or Beclin 1 and has no effect on mTOR activity and the LC3, p62 or Lamp2 protein levels (Supporting Information Fig. 3B and C), we can conclude that the observed phenotypes are due to Vps15 loss of function. Of note, this Vps15 truncation is sufficient to severely blunt starvation-induced puncta formation for two PI3P-binding proteins regulating autophagy, mCherry-WIPI-1 and the endogenous WIPI-2 (Fig. 2A and B). Our data indicate that the N-terminus domain of Vps15 containing the myristoylation signal for membrane localization and the putative catalytic domain is required to regulate Vps34 stability and PI3K activity, and to maintain cell viability. In addition, we demonstrate that in these cells the Vps15/Vps34 complex is critical for the regulation of PI3P-binding FYVE-domain proteins, ruling out a major role of other PI3K classes or PIP phosphatases.

The implication of Vps15/Vps34 complex in mTOR activation in response to nutrients and in particular to amino acids is extremely controversial. The role of Vps34 as an amino acid availability sensor was proposed using Vps34 and Vps15 knockdown by RNAi in human cells (Byfield et al, 2005; Nobukuni et al, 2005). More recently, it has been proposed that Vps34/Vps15 may mediate the effects of amino acids on mTOR through the stimulation of phospholipase D activity and phosphatidic acid production (Yoon et al, 2011). However, genetic studies in drosophila flies and nematodes did not confirm these observations (Avruch et al, 2009; Juhasz et al, 2008). In *Vps34*^−/−^ mouse embryos immunostaining of phosphorylated rpS6 was reduced, suggesting a reduced mTORC1 activity (Zhou et al, 2011). However, since the development and viability of *Vps34*^−/−^ mutants was severely affected, a general reduction in nutrient uptake and sensing, that indirectly leads to the shut-down of the mTOR pathway, should be considered. *In vivo* we do not observe dramatic changes of mTOR signalling in *Vps15*-deficient MEFs and skeletal muscles, as growth factor stimulation is modestly reduced while nutrient stimulation leads to a minor up-regulation (Figs. Fig. 4 and Fig. 5B; Supporting Information Fig. 5). It is conceivable, although unlikely, that the regulation of mTOR activity by Vps34/Vps15 is somehow limited to human cells. Alternatively, the effects of Vps34/Vps15 on mTOR may not be direct, but secondary consequences of cellular responses to the disruption of the Vps34/Vps15 complex. Interestingly, lysosomes and p62 levels are involved in upstream regulation of mTOR activity (Duran et al, 2011; Zoncu et al, 2011), and both components are dramatically increased in Vps15 mutant cells (Fig. 2D and F; Fig. 3B; Fig. 6B and C). Similarly, recent data on conditional *Vps34* knockout mice in liver and heart (Jaber et al, 2012) indicated that the steady-state level of mTOR signalling was not affected in *Vps34*-null MEFs, liver or cardiomyocytes. However, contrary to our observations, amino acid-stimulated mTOR activation was suppressed in the absence of Vps34 *in vitro* in immortalised MEF cultures. One possible explanation for this discrepancy is that we used primary cultures of MEFs to assess the status of mTOR signalling in response to both amino acids and dialysed foetal calf serum. Moreover, we addressed the dynamics of mTOR activation by both stimuli in time course experiments. Finally, we measured wortmannin sensitivity and showed similar sensitivity of control and *Vps15* mutant cells to the PI3K inhibitor, suggesting that additional PI3K classes mediate mTOR activation by amino acids. However, we cannot exclude that *Vps34* deletion causes effects on mTOR signalling that are not mimicked by *Vps15* deficiency.

Different Vps15/Vps34 complexes are involved in multiple steps of autophagosome formation and endosomal trafficking. A complex also containing Beclin 1 and ATG14L promotes autophagosome formation during the early nucleation step, at least in part through the recruitment of the PI3P binding effectors WD repeat domain phosphoinositide-interacting (WIPI) proteins at the phagophore assembly sites (Matsunaga et al, 2009). Moreover, a distinct Vps15/Vps34 complex containing Beclin 1 and UVRAG, and localized at the endosomes, regulates autophagosome maturation by favouring fusion with lysosomes/late endosomes and endocytic trafficking (Zhong et al, 2009). In Vps15 mutant MEFs and skeletal muscles we still detect autophagosome formation as indicated by endogenous LC3 lipidation and puncta formation after LC3-GFP ectopic expression (Fig. 2C, D and E; Fig. 3A and B; Fig. 5A, Fig. 6A, B and D). More importantly, EM of skeletal muscles undoubtedly reveals the accumulation of double membranated autophagosomes containing mitochondria, glycogen and cytosolic components in Vps15 mutant tissue (Figs. Fig. 7 and Fig. 8C; Supporting Information Fig. 10). Our data are consistent with recent analysis of *Vps34*-deficient sensory neurons and lymphocytes reporting defects in the endosomal pathway but not in autophagosome formation (McLeod et al, 2011; Zhou et al, 2011), while they contrast with the reported defects of autophagy initiation in *Vps34*-deficient liver and heart (Jaber et al, 2012). It is possible that in different cell types and physiological conditions, the Vps15/Vps34/Beclin1/ATG14L complex is dispensable for autophagosome nucleation. Interestingly, there is increasing evidence of non-canonical routes to autophagy that can bypass this complex and WIPI-1/2 effectors (Codogno et al, 2011). Conversely, we trace a block in the late phases of the autophagic flux by the following experimental evidences: (i) immunostaining or immunoblot analysis of p62, ubiquitin and lysosomal marker Lamp2, indicating that ubiquitinated cargo proteins are not properly digested after p62-mediated delivery to the lysosomes (Fig. 2D, E and G); (ii) lack of red puncta induction in Vps15 mutant MEFs after ectopic expression of mRFP-EGFP tandem fluorescent-tagged LC3 reporter protein, consistent with an alteration in acidic pH or hydrolase content that does not efficiently quench the EGFP signal (Fig. 3A); (iii) flux experiment with lysosomal inhibitors, showing that mutant cells mimic the effect of bafilomycin A1 on LC3 processing (Fig. 3B and Supporting Information Fig. 3A); (iv) processing defects of GST-BHMT fusion reporter protein at the autolysosome step (Fig. 3C); (v) decreased activity of lysosomal enzymes and sorting defects leading to the excretion to the enzymes to the extracellular fluid (Fig. 3D). These defects in the autolysosomal function suggest that Vps15/Vps34 complexes containing UVRAG or additional partners are impaired by the *Vps15* deletion and are not functionally compensated. Notably, there is growing evidence highlighting the important role of PI3P in the autophagosome maturation. For instance, reduction of cellular PI3P levels by overexpression of PI3P phosphatase MTMR3 results in decreased size of autophagosomes suggesting a role for PI3P in autophagosome maturation (Taguchi-Atarashi et al, 2010). Recently, the essential direct role of TECPR1 protein in autophagosome maturation has been demonstrated (Chen et al, 2012). TECPR1 is a binding partner of Atg5/Atg12 complex which requires PI3P for its function on complex recruitment to autolysosomes. Strikingly, depletion of TECPR1 phenocopies the deletion of *Vps15* in terms of p62 and LC3 accumulation while autophagosome formation is preserved. Since *Vps15*-depleted cells have decreased levels of PI3P, it is tempting to speculate that PI3P are indispensable for autophagosome maturation by acting on target proteins such as TECPR1.

Consistently, the phenotype of *Vps15*-deficient skeletal muscles differs from the Atg7 mutants (Fig. 6; Fig. 8; Supporting Information Fig. 9C and D). In contrast to the Vps15 mutants, *Atg7* deletion impairs LC3 lipidation, autophagosome formation and mitophagy. However, *Atg7* deletion does not cause accumulation of glycogen and sarcolemmal features within the fibres that are observed in the Vps15 mutants. Interestingly, dysfunctional Endosomal Sorting Complex Required for Transport (ESCRT) leads to defects similar to the *Vps15* deletion. The ESCRT machinery consists of four protein complexes, named ESCRT-0, -I, -II, -III, that are involved in sorting of ubiquitinated cargoes and in multivesicular endosome biogenesis (Rusten et al, 2012). Depletion of ESCRT subunits causes autophagosome accumulation and defects in receptor tyrosine kinase recycling (Lee et al, 2007; Rusten et al, 2007), adding new evidence that defects in late endosomal/lysosomal functions may be responsible of the Vps15 mutant phenotype. Whether the autophagosome build-up observed in lysosomal storage disease and ESCRT deficiency is a cause or a compensatory response of the disease state is still an open question. Inhibition of autophagy by Atg5 and Atg7 knockdown delays neuronal cell loss caused by dysfunctional ESCRT-III (Lee and Gao, 2009). However, in an animal model of lysosomal storage Pompe disease due to GAA deficiency, the combined deletion of Atg5 worsens the myopathy, though permitting successful enzyme replacement therapy in autophagy deficient skeletal muscles (Raben et al, 2008; Raben et al, 2010). It will be important to determine whether the myopathy in Vps15 mutants is affected by altering the autophagosome formation.

AVMs are rare disorders including Danon disease and X-linked myopathy with excessive autophagy (XMEA), infantile AVM, adult-onset AVM with multi-organ involvement, X-linked congenital AVM (Nishino, 2003). The only known causative gene is *Lamp2* whose mutations cause Danon disease. In addition, lysosomal storage diseases are metabolic disorders that are often, but not exclusively, due to mutations in genes encoding lysosomal degrading enzymes, leading to defects in lysosomal function (Eskelinen and Saftig, 2009). Individually, AVMs and lysosomal storage diseases occur with incidences of less than 1:100,000. However, as a group the incidence is about 1:5000–1:10,000 (Fuller et al, 2006). Interestingly, in both AVMs and lysosomal storage diseases the accumulation of autophagosomes, glycogen and sarcolemmal features are pathognomonic morphological hallmark of the disease. We show that *Vps15* loss-of-function mouse mutants recapitulate these features of the disease (Fig. 5; Fig. 6; Fig. 7; Fig. 8), while Vps15/Vps34 gain-of-function approaches in human muscle cells from Danon patients alleviate LC3 and glycogen accumulation (Fig. 9C and D). Although future work will tell whether mutations in the Class III PI3K pathway underlie a fraction of human AVM, our study unquestionably demonstrates that the modulation of Vps15/Vps34 expression has a direct functional role in the development of AVM.

## MATERIALS AND METHODS

### Generation of Vps15^f/f^ mice

The Vps15 conditional mutant mouse line was established at the MCI/ICS (Mouse Clinical Institute – Institute Clinique de la Souris, Illkirch, France). The targeting vector was constructed as follows. The 5′ (4.2 kb), 3′ (3.5 kb) and inter-loxP (1.4 kb) fragments were PCR amplified on a 129 BAC DNA (reference bMQ366b03) and sequentially subcloned into an MCI proprietary vector containing the LoxP sites and a Neo cassette flanked by Flippase Recognition Target (FRT) sites. The linearized construct was electroporated in 129S2/SvPas mouse embryonic stem (ES) cells. After selection, targeted clones were identified by PCR using external primers and further confirmed by Southern blot with 5′ and 3′ external probes. Two positive ES clones were injected into C57BL/6J blastocysts, and derived male chimaeras gave germline transmission. The excision of the neomycin-resistance cassette was performed *in vivo* by breeding the chimeras with a Flp deleter line (C57BL/6N genetic background). The Flp transgene was segregated by breeding the first germ line mice with a wild type C57BL/6N animal. For generation of whole body or muscle specific *Vps15* knockout, Vps15 floxed mice were crossed with transgenic mice expressing Cre recombinase under the control of a CMV promoter or Human Skeletal Actin promoter, respectively (Miniou et al, 1999; Schwenk et al, 1995). Genomic DNA isolated from mouse tail snip or tissues was analysed by PCR. Cre-mediated recombination was confirmed by PCR with genomic DNA from muscles. The following PCR primers were used:

EF 5′-GCTAGGCCCTCTTAGACGGTTTCAGAC-3′;
ER 5′-AGCTGTGTGCTTCTGTAGCAGCAACTG-3′;
LF 5′-GACCGAGGCATACGGTACTTTTACG-3′;
LR 5′-ACGTCATGTCATTCTTTCCAGCCGC-3′.

The following combinations of the primers were used: to check presence of distal loxP sites—LF/LR; to check Cre mediated excision of the locus—LF/ER; to check Flp mediated excision of Neomycin cassette—EF/ER. The primers used for Cre recombinase are: 5′-GCGGTCTGGCAGTAAAAACTATC-3′ and 5′-GTGAAACAGCATTGCTGTCACTT-3′. All animal studies were approved by the Direction Départementale des Services Vétérinaires, Préfecture de Police, Paris, France (authorization number 75-1313).

### Knockout mice and *in vivo* transfection experiments

Muscle specific *Atg7* knockout mice were described previously (Masiero et al, 2009). *In vivo* transfection experiments were performed by intramuscular injection of plasmid DNA in TA muscle followed by electroporation as described (Mammucari et al, 2007). Muscles were collected 10 days after electroporation and frozen in liquid nitrogen for subsequent analyses. Cryosections of TA muscles expressing YFP-LC3 were examined in a fluorescence microscope as described (Mizushima et al, 2004). Lamp2 knockout mice were described previously (Tanaka et al, 2000).

### Reagents

The following primary antibodies were used: anti-Vps15 antibody and anti-p62 (SQSTM) (Abnova), anti-LAMP2 and anti-LAMP1 (Abcam), anti-Acetylcholinesterase (Novus), anti-Dystrophin (Novocastra), anti-WIPI-2 (Abgent), anti-Atg14L (MBL). All other antibodies were from Cell Signalling. 2xFYVE-GFP expressing vector was kindly provided by Sharon Tooze. EGFP-LC3 adenovirus was a kind gift of Aviva Tolkovsky (49), tfLC3 adenovirus was a kind gift from Junichi Sadoshima (Hariharan et al, 2010). The human WIPI-1 cDNA was subcloned from pEGFP.C1-WIPI-1 (Proikas-Cezanne et al, 2004) into EcoRI/XhoI of mCherry (kindly provided by Roland Brock). GST-BHMT construct was a kind gift from Carol Mercer (Dennis et al, 2009). Vps15, Vps34 and ΔVps15(293-1358 aa) adenoviruses were generated by Vector BioLabs (USA). Wortmannin and bafilomycin A1 were from Sigma.

### Cell cultures

Vps15^f/f^ MEFs were prepared from embryos at embryonic day 13.5 as previously described (Shima et al, 1998). Briefly, embryos were minced and incubated in 0.25% trypsin at 37°C for 15 min, then passed through a cell strainer (Falcon). Dulbecco's modified Eagle's medium (DMEM), containing 10% of fetal calf serum (FCS) was added to the cell suspension. Cells were then centrifuged at 1000 rpm and the pellet was suspended in DMEM containing 10% FCS. To obtain Vps15^−/−^MEFs, fibroblasts were transduced at 50 MOI by adenoviruses Cre-GFP or GFP as a control and incubated for 3 days before analyses.

For LC3 analyses MEFs were transduced with LC3 adenoviruses (EGFP-LC3 or tfLC3) 48 h after CAG-Cre adenovirus transduction. For FYVE domain and WIPI-1 analyses MEFs were transfected with 2xFYVE-GFP or mCherry-WIPI-1 using Lipofectamine 2000 reagent as recommended by manufacturer (Invitrogen).

For growth factor or amino acid treatment experiments MEFs were first starved for 18 h in serum-free medium and then additionally for 2 h in EBSS. Cells were then stimulated for indicated time with dialysed Fetal bovine serum (FBS) (final concentration 10%) or stimulated with amino acid mixture. After stimulation, the final concentration of amino acids in the media was the same as in DMEM. The 10× amino acid mixture was prepared from individual amino acid powders.

GST-BHMT assay was performed as described previously (Dennis et al, 2009).

Primary myoblast cultures were obtained from gastrocnemius and TA muscles of 4-week-old mice, as described (Aguilar et al, 2007). Briefly, muscles were digested with five sequential 10-min incubations in DMEM/HamF12 medium containing 0.14% pronase (Sigma). The supernatants from the second, third and fourth digestions were pooled and filtered through a 100 µm cell strainer. Cells were centrifuged, washed twice, counted and plated at low density (100 cells/cm^2^) on 12-well plates coated with gelatin (Type A from pig skin; Sigma). Cells were grown in medium composed of DMEM/Ham F12, 2% Ultroser G (Biosepra), 20% FCS, penicillin, streptomycin and l-glutamine. After 1 week, wells containing myoblasts without contamination with fibroblasts were trypsinized, pooled and expanded. For the experiments, myoblasts were differentiated to myotubes in DMEM/Ham's F12 medium supplemented with 2% horse serum. At day 2 of differentiation myotubes were transduced by Cre adenovirus at 5MOI and incubated for another 3 days for immunoblot analyses and 2 days before transduction with EGFP-LC3 adenovirus. The subcellular distribution of EGFP-LC3 was analysed 24 h post-infection by fluorescent microscopy.

In rescue experiments *Vps15*-deleted and control MEFs were infected with GFP, Vps15, Vps34 or ΔVps15 adenoviruses at 200 MOI and analysed 60 h after.

Human primary myoblasts were established from muscle obtained from biceps brachii (Nishino et al, 2000). Genetic mutations were identified for 02-74M (Danon disease) LAMP2 c.928G > A and for 08-967 (Danon disease) LAMP2 c.371_372del. Cells were grown and differentiated similarly to mouse myoblasts. Two days after differentiation cells were transduced by GFP, Vps15, Vps34 or ΔVps15 adenoviruses at 150 MOI. Three days post-infection cells were analysed by immunoblot or stained with PAS to visualize glycogen.

### Metabolic studies in mice

At two months of age, a GTT was performed in mice after an overnight fasting (14 h). Mice were intraperitoneally injected with 2 g/kg glucose, and blood was collected from the tail vein for determination of glucose levels at 0, 15, 30, 45 and 100 min by Glucotrend glucometer (Roche Diagnostics). ITT was performed at two months of age. Overnight fasted mice were intraperitoneally injected with 1 U/kg insulin (Actrapid) and the glucose concentration in whole blood from the tail vein was measured at 0, 15, 30 and 60 min.

### Histology

Soleus, EDL and TA muscles of male mice were embedded in Cryo-Gel (Electro Microscopy Sciences), frozen in isopentane pre-chilled in liquid nitrogen and stored at −80°C. Transverse 6 µm thick cross sections were collected along the entire length of the muscle at 300 µm intervals with a cryostat (Leica CM 1850) and stained with haematoxylin/eosin solution, PAS or assayed for COX activity.

### Microscopy

For confocal microscopy cells were grown on coverslips, fixed with 4% paraformaldehyde in PBS and permeabilized with 0.2% TritonX100 in PBS for 5 min, followed by blocking in 3% BSA in PBS. Slides were then treated with primary antibodies overnight. Secondary antibodies used for this assay were anti-rat IgG Alexa Fluor 488 and anti-mouse IgG Alexa Fluor 555 (Invitrogen). Confocal images were acquired with an optical slice of 0.8 µm using a 40×/0.75 oil immersion objective using LSM 700 confocal microscope (Zeiss) and analysed using ZEN software (Zeiss).

Fluorescence and light microscopy were performed using an inverted microscope (Eclipse Ti-S; Nikon) and 10×/0.30, 20×/0.50 or 40×/0.785 Plan Fluor objectives (Nikon). Images were captured using a Super high-definition cooled colour camera head DS-Ri1 (Nikon) and NIS Elements software (Nikon). All samples for microscopy were viewed at room temperature.

Direct fluorescence of mCherry-WIPI-1 and indirect immunofluorescence of endogenous WIPI-2 was conducted as previously described (Proikas-Cezanne et al, 2004; Proikas-Cezanne and Pfisterer, 2009). Z-stacks (optical sections of 0.5 µm) were acquired using Zeiss/Axiovert 100M/LSM510 and a 63 × 1.4 DIC Plan-apochromat objective, and projections of individual optical sections were used to generate final images. mCherry-WIPI-1 puncta formation was assessed as previously described (Proikas-Cezanne et al, 2007).

### Protein extraction, immunoblotting and immunoprecipitation

For immunoblot analysis, cells were washed twice with cold phosphate-buffered saline (PBS), scraped off of the culture dish in lysis buffer B containing 50 mM Tris (pH 8.0), 1% NP-40, 120 mM NaCl, 20 mM NaF, 1× protease inhibitors (Roche), 1× PhosphoStop Inhibitors (Roche). Protein extract from muscle tissue was prepared in 20 mM Tris-HCl (pH 8.0), 5% glycerol, 138 mM NaCl, 2.7 mM KCl, 1% NP-40, 20 mM NaF, 5 mM EDTA, 1× protease inhibitors (Roche), 1× PhosphoStop Inhibitors (Roche). Homogenates were spun at 12,000×*g* for 10 min at 4°C. Protein extracts were resolved by SDS-PAGE before transfer onto PVDF membrane and incubation with the primary antibodies. For immunoprecipitations, the indicated antibodies were added to the cell extracts and incubated at 4°C for 2 h. The complexes were pulled down by incubating at 4°C for additional 1 h with protein G-sepharose (GE Healthcare). The resulting beads were washed with lysis buffer five times and complexes eluted by boiling in sample buffer before proceeding with immunoblot analysis.

### Real-time quantitative PCR

Total RNA was isolated using RNAeasy Mini Kit (QIAGEN), single-strand cDNA was synthesized from 1 µg of total RNA with random hexamer primers and SuperScript II (Invitrogen). Real-time quantitative PCR (RTqPCR) was performed on MX3005P instrument (Agilent) using a Brilliant II SYBR Green QPCR Low ROX Master Mix (Agilent). The relative amounts of the mRNAs studied were determined by means of the 2^−ΔΔCT^ method, with pinin as a reference gene and control treatment or genotype as the invariant control. Pairs of primers were used in the study: *Vps15* exon 2 CCTGGTGGTTGTGAAGGTCT and AGCGCTTCTCGATGTTGTTT, *Vps15* exon 4 TTGTCCTGGTGTCCGTGATA and GAGTGTCCTCAGGGCTTCAG, TNF-α CAAGGGACAAGGCTGCCCCG and GCAGGGGCTCTTGACGGCAG, IL6 TAGTCCTTCCTACCCCAATTTCC and TTGGTCCTTAGCCACTCCTTC, IL1β CTGGTGTGTGACGTTCCCATTA and CCGACAGCACGAGGCTTT, F4/80 CCCCAGTGTCCTTACAGAGTG and GTGCCCAGAGTGGATGTCT. For analyses of TFEB and its targets transcript levels, primer sequences were taken from (Pena-Llopis et al, 2011).

### Biochemical assays

For glycogen content, frozen tissue samples were powdered in liquid nitrogen. The powder was used for enzymatic analysis of glycogen after perchloric acid extraction (Bergmeyer and Bernt, 1974). For creatine kinase assay, blood samples were taken by peri-orbital bleeding in random-fed mice. Activity of creatine kinase in plasma was determined with a multiparametric automate (Olympus AU 400) by colorometric density.

Lysosomal enzyme activities were measured using fluorometric assays. Cell lysates were prepared in water by repetitive freezing-defrosting cycles. Then 20 µL of cell culture media or 3 µg of total protein extract were incubated at 37°C with 0.1 mL of 0.1 M sodium citrate, pH 4.6 containing the corresponding 4-methylumbelliferyl substrate (3 mM). The reaction was stopped with 0.1 mL 0.2 M glycine, pH 10.4, and the 4-methylumbelliferyl fluorescence was measured using fluorometric plate reader.

**The paper explained**

PROBLEM:

Autophagy plays an important role in tissue homeostasis and renewal through the isolation of cellular components and their disposal in lysosomes. The relevance for skeletal muscle function is underlined by the observation that a variety of human myopathies are accompanied by a deregulation of autophagic flux. The Vps15/Vps34 complex has Class III PI3 kinase activity. Genetic studies in yeast have demonstrated the Vps15/Vps34 requirement for endosomal sorting and autophagy. However, the pathophysiological impact of these gene products in mammalian tissues, including skeletal muscle, remains unclear and is the object of our investigation.

RESULTS:

Vps15 loss-of-function mutants in skeletal muscles recapitulate a disease condition of AVM, characterized by profound muscle weakness and the accumulation of autophagosomes, glycogen and sarcolemmal features within the muscle fibres. Conversely, the overexpression of Vps15 and Vps34 in human muscle cells from Danon patients suffering of AVM alleviates metabolic disturbances.

IMPACT:

We have characterized *in vivo* the steps in autophagosome formation and endosomal trafficking that require Class III PI3K activity in mammalian cells. Our findings in human patient cells and animal model open the possibility that the modulation of Vps15/Vps34 signalling has an important functional role in the development of AVMs and lysosomal storage diseases. Finally, strategies ameliorating Vps15/Vps34 functions may be beneficial for this broad class of metabolic diseases.

### Electron microscopy

EDL muscles were dissected from Vps15^f/f^ and HSA-Cre^+^;Vps15^f/f^ mice. Muscles were fixed at RT in 3.5% glutaraldehyde in 0.1 M sodium cacodylate buffer (pH 7.4) for 2 h and kept in fixative before further use. In standard embedding preparations, small bundles of fixed fibres were post-fixed in 2% OsO_4_ in 0.1 M sodium cacodylate buffer for 2 h and block-stained in aqueous saturated uranyl acetate. For glycogen analysis, small bundles of fixed fibres were post-fixed in 0.5% OsO_4_-1.5% Fe(CN)_6_. Specimens from both procedures, after dehydration, were embedded in an epoxy resin (Epon 812). After staining with Toluidine Blue dye, the sections were viewed on a Leica DMLB fluorescence microscope (Leica Microsystem). For EM, ultrathin sections (50 nm) were cut and, after staining in 4% uranyl acetate and lead citrate, examined with a Morgagni Series 268D electron microscope (FEI Company), equipped with Megaview III digital camera.

### *In vitro* Vps34 lipid kinase assay

Immuno-purified complexes were washed in 1× kinase base buffer (KBB) consisting 20 mM HEPES pH 7.4, 1 mM EGTA, 0.4 mM EDTA, 5 mM MgCl_2_. Half of the volume was taken for input, the remainder was centrifuged and excess 1× KBB was removed. 40 µL of 1× kinase assay buffer was added to precipitated kinase (1×KBB supplemented with 0.1 mg/mL phosphatidylinositol, 50 µM cold ATP, 5 µCi ^32^P-ATP, 5 mM MnCl_2_ and 50 µM DTT) followed by incubation at 37°C for 30 min with vigorous shaking. Reaction was quenched by addition of 1 M HCl, followed by lipid extraction with two volumes of MeOH:CHCl_3_ (1:1). Organic phase was resolved by thin layer chromatography (Whatman). Resolution of phospho-lipids was achieved using a buffer composition of CHCl:MeOH(99%):NH_4_OH(30%):water (129:100:4.29:24). Resolved plates were analysed by autoradiography. Vps34 was pharmacologicaly inhibited by addition of 50 nM Wortmannin in cell culture and kinase reaction buffer.

### Statistical analysis

A two-tailed Student's *t*-test was used for statistical analysis. All data are expressed as means ± SEM, and significance was established at the *p* ≤ 0.05 level.

## Supplementary material

As a service to our authors and readers, this journal provides supporting information supplied by the authors. Such materials are peer reviewed and may be re-organized for online delivery, but are not copy-edited or typeset. Technical support issues arising from supporting information (other than missing files) should be addressed to the authors.
