# Supplementary material for: Defects of Vps15 in skeletal muscles lead to autophagic vacuolar myopathy and lysosomal disease
Source: EMBO Mol Med. 2013 Apr 30;5(6):870–90. doi: 10.1002/emmm.201202057 (PMC3779449; doi:10.1002/emmm.201202057)
Supplement: Supplementary file 1 [file emmm0005-0870-SD1.pdf]

EMBO Molecular Medicine

Defects of Vps15 in skeletal muscles lead to autophagic vacuolar myopathy and lysosomal disease

Ivan Nemazanyy, Bert Blaauw, Cecilia Paolini, Catherine Caillaud, Feliciano Protasi, Amelie Mueller, Tassula Proikas-Cezanne, Ryan C. Russell, Kun-Liang Guan, Ichizo Nishino, Marco Sandri, Mario Pende, Ganna Panasyuk

Corresponding author: Mario Pende, INSERM

Review timeline:

Submission date:	20 September 2012
Editorial Decision:	13 November 2012
Revision received:	21 February 2013
Accepted:	13 March 2013

Transaction Report:

(Note: With the exception of the correction of typographical or spelling errors that could be a source of ambiguity, letters and reports are not edited. The original formatting of letters and referee reports may not be reflected in this compilation.)

Editor: Roberto Buccione

1st Editorial Decision 13 November 2012

Thank you for the submission of your manuscript to EMBO Molecular Medicine.

We are very sorry for the delay in getting back to you with the Reviewers' evaluation on your work. Unfortunately, in this case we experienced unusual difficulties in securing three appropriate reviewers in a timely manner.

While Reviewer 1, who unfortunately delivered a delayed report in this case, was generally positively disposed, both Reviewer 2 and 3 raised major and consistent issues. Hence, although the topic of the study is felt to be potentially interesting, a number of substantial concerns prevent us from considering publication at this time.

Reviewer 2, while recognizing the relevance of links between Vps15 deficiency and human myopathies, suggests that it would have been more informative, given the focus on skeletal muscles, to show the phenotype of Vps15-deficient mouse embryo fibroblasts (MEFs) converted into skeletal muscle by MyoD. S/he also feels that the analysis of muscle histology should include additional parameters.

Reviewer 3 is concerned that the relationship between Vps15 defects and lysosomal diseases in not clear and expresses doubts about the potential clinical relevance of your findings.

Both Reviewer 2 and 3, are especially concerned with your observation that the Vps15 null MEFs obtained after Cre recombination actually express a truncated 100Kd product, which may have

unexpected consequences. Data would need to be presented that definitively evaluates the contribution of the truncated Vps15 to the phenotypes described.

While it is clear that publication of the paper cannot be considered at this stage, I am open to the submission of a substantially revised manuscript, provided however, that the Reviewers' concerns are fully addressed with additional experimental data where appropriate.

I understand that the amount of work that would be required is significant and I would perfectly understand if you decided to submit your work elsewhere. I should remind you that it is EMBO Molecular Medicine policy to allow a single round of revision only and that, therefore, acceptance or rejection of the manuscript will depend on the completeness of your responses included in the next, final version of the manuscript.

As you know, EMBO Molecular Medicine has a "scooping protection" policy, whereby similar findings that are published by others during review or revision are not a criterion for rejection. However, should you decide to submit a revised version, I do ask you to get in touch with us after three months if you have not completed it, to update us on the status. Please also contact us as soon as possible if similar work is published elsewhere.

***** Reviewer's comments *****

Referee #1 (General Remarks):

This is a very complete and thoughtful study, in line with prior work on the VPS34 null animals, providing new insights into mechanisms of autophagy-related muscle pathology.

There are just a few things for the authors to work on :

1) Figure 4; while it's clear mTOR signaling isn't decreased, I'm not sure I'd say it's even slightly increased based on tis data.

2) Figure 5; given that muscle is syncytial, and thus multi-nuclear, it's not surprising to see some remaining Vps15 after recombination via the HSA Cre. Therefore please delete the speculation that the Vps15 is from other sources, since this is not proven... and it's likely still from skeletal muscle. (this does not detract from the findings).

3) Figure 8; there's something of a correlation/causation issue here. Even though Vps15 deletion causes a phenotype reminiscent of vacuolar myopathy (AVM), it seems incorrect to imply this is causative of the actual AVM, given the mutations that cause that disease are known. Also the gain of function data does not prove this - but rather proves that activation of growth factor pathways can fight autophagy, which is not terribly surprising. So perhaps the conclusions here can be softened a little.

4) Figure 8F; there are no statistics given for the functional (strength) data, either in the figure or in the figure legends. Were the changes statistically significant?

5) The paper needs another edit for English. There are many many instances of dropped articles and other issues.

But in all, this is a very valuable and complete study.

Referee #2 (Comments on Novelty/Model System):

It would way more informative to show the phenotype of Vps15-deficient MEFs converted into skeletal muscle cells by MyoD.

Moreover, the histological analysis of muscles from Vps15 mutant mice should be expanded by

measuring parameters of inflammatory infiltrate, necrosis, cross sectional area (CSA) with appropriate tools.

Referee #2 (General Remarks):

In this manuscript Nemazanyy et al. investigated the function of Vps15 in skeletal muscles by generating Vps15 deficient mouse embryonic fibroblasts (MEF) and mouse with skeletal muscle specific deficiency of Vps15, and performing an analysis on several aspects of autophagosome formation and activity, as well as parameters of muscle function. The results from both in vitro and in vivo studies reveal common features observed in consequence of Vps15 deficiency, including defective autophagy maturation, lysosome function and glycogen storage, ultimately leading to a phenotype reminiscent of human autophagic vacuolar myopathy. A comparative analysis reveals clear differences between Vps15 and Atg7 mutant mice. Moreover, the authors report on the unexpected activation of mTOR in Vps15 mutant cells.
Overall, this work provides a large amount of data on the consequence of Vps15 deficiency; thus, despite being diffuse and descriptive, the manuscript is informative and suggests interesting links between Vps15 deficiency and human myopathies. As such, I believe it is in principle of interest for EMBO Molecular Medicine.

Specific comments:

The authors document the presence of a truncated form of Vps15 as result of their gene targeting strategy. However, they do not provide any data on the potential function and localization of this truncated product in Vps15 deficient MEFs and mice. I suggest doing so, by monitoring by IF the expression pattern and localization of this truncated proteins using the same antibody that recognizes the 100 KD band by WB). Likewise, the authors should compare the effect of Vps15 WT vs 100 KD form in the overexpression experiments shown in the manuscript.

The focus of the manuscript is clearly of Vps15 function in skeletal muscles. For this reason it would way more informative to show the phenotype of Vps15-deficient MEFs converted into skeletal muscle cells by MyoD.

The histological analysis of muscles from Vps15 mutant mice should be expanded by measuring parameters of inflammatory infiltrate, necrosis, cross sectional area (CSA) with appropriate tools.

Referee #3 (General Remarks):

The paper by Nemazanyy et al "Defects of Vps15 in skeletal muscle lead to autophagic vacuolar myopathy and lysosomal disease" describes the effect of Vps15 deficiency in cells, in the whole mouse, and in murine skeletal muscle. Each experiment is very well designed, performed using state-of-the-art techniques, and nicely presented. However, the major problem with the paper is that the overall story connecting the experiments with a clear rationale and conclusion is not defined.

The experiments seem to have been done for the sake of doing experiments-there is no clear explanation as for why the authors chose to focus on the role of Vps15 in general, and why they chose to focus on the role of Vps15 in skeletal muscle. Furthermore, although the results of the experiments turned out to be unexpected and not uninteresting, the implications are not clear. The myopathy which they created does have some common features with known autophagic myopathies, but it is not clear how this "new myopathy" will help to understand the pathogenesis of any of the "real" diseases. Along the same lines, it is stated in the title that Vps15 defect leads to "lysosomal disease". Here again, it is not clear if there is any connection with any known lysosomal disorder. If the authors mean that they unexpectedly created a model of Danon disease, they should say so, and they should explain what the advantages of this model.

Specific Comments

The authors should clearly indicate why they chose skeletal muscle for tissue-specific deletion of

Vps15.

The term "Vps15-null" is misleading since a 100kDa product is made after Cre recombination. Importantly, the presence of this product is very disturbing, because this protein may well be a culprit responsible for the unexpected results. At least, one can not rule this out.
In addition, it is not clear if this truncated protein is made in skeletal muscle of muscle-specific KO mice: Figure 5A does not show any lower band in the KO. Please clarify this point. Another problem with muscle specific KO is a great variability between different mice/samples (i.e., for the Vps15 bands in Fig 5A)? Please comment more on this, beyond simply mentioning incomplete excision.

The paper claims that there is an increase in the number of lysosomes. What is the mechanism for the stimulation of lysosomal biogenesis (Figure 6 C)? This deserves some comments. And again, what if the stimulation of the lysosomal biogenesis is a function of the 100 kDa protein? Given the increase in the number of lysosomes and autophagosomes, what is the mechanism of incomplete flux?

In Vps15-deficient skeletal muscle, glycogen is shown to accumulate in the cytosol and in autophagosomes. Does glycogen accumulate in lysosomes? Does deletion of Vps15 in muscle mimic Pompe disease? Is this what the reference to "lysosomal disease" in the title refers to?

In Figure 9A, the authors analyze the effect of Vps15 over-expression in Vps15-depleted MEFs. It is not clear why this over-expression would increase the amount of 100 kDa product in the Cre condition but not in the GFP (control) condition.
From these experiments, it looks like over-expression of Vps15 in Vps15-depleted MEFs does not affect the level of Vps34 but still affects p62 (Fig 9A), suggesting that Vps34 is not important for this phenomenon. If indeed Vps15 overexpression alone was sufficient to have an effect on p62, what is the reason for using transfection with Vps15 and Vps34 combined for human myoblasts (Fig 9B)? The use of the combination of Vps15 and Vps34 needs to be justified.

It looks like Vps15 is an abundant protein in MEFs and in skeletal muscle but not in human myoblasts (in either control or in Danon disease; compare Fig 5A, 9A-B). The authors need to elaborate.

Minor comments:

It would be nice to provide a reference for the lysosomal storage disease incidences described on page 20.

Italics or no italics on "et al" for citations (e.g., page 3)

Page 20: The content for the reference to Atg7KO is not correct; the claim that Atg7 KO on Pompe background worsens phenotype is not accurate.

The reference "Yan and Becker, 2007" (page 7) does not seem to correspond to the role of Vps15 in stability of the complex.

Page 10: methyltransferase missing "n" (type-o)

1st Revision - authors' response 21 February 2013

We thank the three reviewers and we appreciate they found specific interests in our study. In this revised version, we provide new functional data addressing the impact of Vps15/Vps34 activity on lysosomal function and autophagic vacuolar myopathy. Briefly, we address three main points. First, we show that deletion of the N-terminal domain of Vps15 results in a complete loss of function mutant. Second, we reveal important trafficking defects upon Vps15/Vps34 deletion that lead to mistargeting of lysosomal enzymes to the extracellular space and contribute to lysosomal

dysfunction. Third, we clarify the relationship between Vps15 and autophagic vacuolar myopathy. In addition, we performed a deeper analysis of signal transduction, muscle cell biology and histology as suggested by the reviewers. There is a large amount of new data that are included in the 9 figures, and 14 supplementary figures of this revised manuscript. Below is our response to the reviewer comments

Referee #1 :
This is a very complete and thoughtful study, in line with prior work on the VPS34 null animals, providing new insights into mechanisms of autophagy-related muscle pathology.
There are just a few things for the authors to work on:
1) Figure 4; while it's clear mTOR signaling isn't decreased, I'm not sure I'd say it's even slightly increased based on this data.

We thank the reviewer for his/her appreciation of our work. We agree that the main message about mTOR signaling is that *Vps15*-deficient cells do not display a major impairment of mTOR activity and maintain wortmannin sensitivity (Figure 4). Although we reproducibly see a trend towards increased mTOR activity in Vps15 deficient cells after amino acid stimulation, we followed the reviewer suggestion and decided not to mention this difference in the revised version because the effects are small and would dilute the message.

2) Figure 5; given that muscle is syncytial, and thus multi-nuclear, it's not surprising to see some remaining Vps15 after recombination via the HSA Cre. Therefore please delete the speculation that the Vps15 is from other sources, since this is not proven... and it's likely still from skeletal muscle. (This does not detract from the findings).

In the new Supporting information fig. 7 as recommended by the second reviewer, we reveal the infiltration of macrophages and immune T cells in the *Vps15*-deficient muscles. We agree with the reviewer that we do not want to establish how much residual Vps15 is coming from muscle or non-muscle cells, and we, therefore, shortened the text accordingly.

3) Figure 8; there's something of a correlation/causation issue here. Even though Vps15 deletion causes a phenotype reminiscent of vacuolar myopathy (AVM), it seems incorrect to imply this is causative of the actual AVM, given the mutations that cause that disease are known. Also the gain of function data does not prove this - but rather proves that activation of growth factor pathways can fight autophagy, which is not terribly surprising. So perhaps the conclusions here can be softened a little.

In the discussion of this revised version we clarify the cause-effect relationship between Vps15 function and myopathy, as suggested by both reviewers 1 and 3. Our study demonstrates two issues that we think are novel:
i) A mouse model of Vps15/Vps34 deficiency in skeletal muscles causes AVM, a phenotype different from autophagy deficient mutants Atg5 and Atg7.
ii) Gain of function approaches up-regulating Vps15/Vps34 function ameliorates signs of disease in AVM human cell lines.

Both issues point to the novel role of Class III PI3K in AVM pathophysiology, taking advantage of functional approaches. We agree with both reviewers and we certainly do not want to insinuate that we found mutations of Vps15/Vps34 genes in humans causing AVM. Although it is possible that a fraction of orphan AVMs might be directly caused by mutations in elements of the Class III PI3K pathway, this is not the subject of our present study and should be tested in the future.

Whether Class III PI3K mediates growth factor signaling is debated in the literature, and is one of the goals of our study. A recent publication by the Guan group demonstrates that glucose starvation positively or negatively regulates Class III PI3K activity depending on the complex composition (Kim et al., Cell, 2013, 152, 290). Our data do not support an involvement of Class III PI3K in sensing nutrients and growth factors, and controlling mTOR activity in mouse embryonic fibroblasts and skeletal muscles (Fig. 4, 5B, Supporting information Fig. 5). In this revised version, in collaboration with the Guan group, we assay PI3K activity in Vps34-, Atg14L-, Beclin1-containing complexes and demonstrate that they are devoid of kinase activity upon deletion of Vps15 (new Fig. 9A and data not shown). Thus, in a model of functional deficiency in Vps15/Vps34 activity, the growth factor signaling does not seem to be affected and remain sensitive to wortmannin inhibition (Fig. 4C). The amelioration of AVM signs when Vps15/Vps34 is overexpressed is likely due to a direct action on lysosomal function and endosomal trafficking. This represents a rather novel finding, as there are not many examples of improving autophagic flux and clearing lysosomal accumulation of undigested material by genetic means.

4) Figure 8F; there are no statistics given for the functional (strength) data, either in the figure or in the figure legends. Were the changes statistically significant?
The differences observed in force measurements are statistically significant with n=5 and $p \leq 0.05$. Figure legends and figures were modified accordingly (modified Fig. 8F and 8G), we apologize for the omission in the previous version.
5) The paper needs another edit for English. There are many instances of dropped articles and other issues.
We apologize. The revised manuscript underwent an additional round of editing.

Referee #2
It would way more informative to show the phenotype of Vps15-deficient MEFs converted into skeletal muscle cells by MyoD.
Moreover, the histological analysis of muscles from Vps15 mutant mice should be expanded by measuring parameters of inflammatory infiltrate, necrosis, cross sectional area (CSA) with appropriate tools.
In this manuscript Nemazanyy et al. investigated the function of Vps15 in skeletal muscles by generating Vps15 deficient mouse embryonic fibroblasts (MEF) and mouse with skeletal muscle specific deficiency of Vps15, and performing an analysis on several aspects of autophagosome formation and activity, as well as parameters of muscle function. The results from both in vitro and in vivo studies reveal common features observed in consequence of Vps15 deficiency, including defective autophagy maturation, lysosome function and glycogen storage, ultimately leading to a phenotype reminiscent of human autophagic vacuolar myopathy. A comparative analysis reveals clear differences between Vps15 and Atg7 mutant mice. Moreover, the authors report on the unexpected activation of mTOR in Vps15 mutant cells.
Overall, this work provides a large amount of data on the consequence of Vps15 deficiency; thus, despite being diffuse and descriptive, the manuscript is informative and suggests interesting links between Vps15 deficiency and human myopathies. As such, I believe it is in principle of interest for EMBO Molecular Medicine.
Specific comments:
1) The authors document the presence of a truncated form of Vps15 as result of their gene targeting strategy. However, they do not provide any data on the potential function and localization of this truncated product in Vps15 deficient MEFs and mice. I suggest doing so, by monitoring by IF the expression pattern and localization of this truncated proteins using the same antibody that recognizes the 100 KD band by WB. Likewise, the authors should compare the effect of Vps15 WT vs 100 KD form in the overexpression experiments shown in the manuscript.

First of all, we would like to thank the reviewer for highlighting the novelty and importance of our study in order to better understanding the function of Class III PI3K complex in cells and its relevance to muscle pathophysiology.
The concern about the potential contribution of the truncated Vps15 product in the observed phenotypes of the Vps15 mutant cells has also been raised by the reviewer #3. In this revised version we took a variety of approaches to demonstrate that our targeting strategy results in a null mutant and that the truncated Vps15 version does not signal to downstream targets.
i) We have developed adenoviral vector overexpressing truncated Vps15 protein. As suggested by the reviewer we have performed a number of additional experiments to address the outcomes of the overexpression of truncated Vps15 in MEFs and Human Myotubes (new Supporting Information Fig. 3B, 13, 14B and C). In sum, we do not observe that overexpression of truncated Vps15 mimics the effects of our Vps15 targeting strategy. As seen in the new Supporting information fig. 3B, overexpression of truncated Vps15 in wild type cells does not affect levels of p62, LC3 or Lamp2. This overexpression does not change phosphorylation status of downstream mTORC1 targets (S6K and S6) (Supporting Information Fig. 3B). As assessed by 2XFYVE-GFP localization, truncated Vps15 does not alter PI3P levels in both wild type and Vps15 mutant cells (Supporting Information Fig. 13B). Unfortunately, when we attempted the immunostaining with different commercially available anti-Vps15 antibodies, we could not reveal any specific signal as overexpression of full length Vps15 by adenoviral transduction in MEFs did not lead to an increased fluorescence signal (data not shown). Finally, we asked whether overexpression of truncated Vps15 protein could modify the phenotype observed in Danon disease patient myotubes, including glycogen and LC3

accumulation. As shown in the new Supporting information fig. 14B and C, we do not observe significant differences in LC3 protein levels and glycogen accumulation in cells overexpressing truncated Vps15 as compared to control GFP expressing cells.
ii) We have provided additional experiments to demonstrate that our targeting strategy causes a complete loss of Class III PI3K signaling. We have analyzed PI3K activity in ATG14L, Vps34, Beclin 1 immunoprecipitates and we have observed a loss of PI3P kinase activity in cells depleted of Vps15 (new Fig. 9A and data not shown). In addition, we asked whether the truncated Vps15 could form a complex with other components of the Class III PI3K complex, such as Beclin1 and Vps34. As presented in Supporting Information Fig. 3C, we could not detect truncated Vps15 protein in immunoprecipitates of anti-Beclin1 and anti-Vps34 antibodies.

2) The focus of the manuscript is clearly of Vps15 function in skeletal muscles. For this reason it would way more informative to show the phenotype of Vps15-deficient MEFs converted into skeletal muscle cells by MyoD.
We thank the reviewer for raising this important point. To be as close as possible to cultured muscle cells, in this revised version we prepared primary myoblasts from Vps15 floxed muscles, differentiated them to myotubes in culture and transduced with adenoviral Cre recombinase. In the new Supporting Information Fig. 9, we reproduce in these cells all the observations on PI3K complex stability, autophagy block, mTOR activity that were previously made in MEFs and skeletal muscle tissue.

3) The histological analysis of muscles from Vps15 mutant mice should be expanded by measuring parameters of inflammatory infiltrate, necrosis, cross sectional area (CSA) with appropriate tools.
To address this point, we include staining for markers of macrophages (F4/80) and T cells (CD3) in Tibialis muscle (new Supporting Information Fig. 7A). Additionally, we perform expression studies by QRTPCR and demonstrate a significant increase in proinflammatory cytokines and inflammatory cell markers in Vps15 mutant tissues at the transcript level (new Supporting Information Fig. 7B). The measurements of cross section area and muscle fiber typing are reported in Supporting Information Fig. 6A and B.

Referee #3 :
The paper by Nemazanyy et al "Defects of Vps15 in skeletal muscle lead to autophagic vacuolar myopathy and lysosomal disease" describes the effect of Vps15 deficiency in cells, in the whole mouse, and in murine skeletal muscle. Each experiment is very well designed, performed using state-of-the-art techniques, and nicely presented. However, the major problem with the paper is that the overall story connecting the experiments with a clear rationale and conclusion is not defined.
1) The experiments seem to have been done for the sake of doing experiments-there is no clear explanation as for why the authors chose to focus on the role of Vps15 in general, and why they chose to focus on the role of Vps15 in skeletal muscle. Furthermore, although the results of the experiments turned out to be unexpected and not uninteresting, the implications are not clear. The myopathy which they created does have some common features with known autophagic myopathies, but it is not clear how this "new myopathy" will help to understand the pathogenesis of any of the "real" diseases. Along the same lines, it is stated in the title that Vps15 defect leads to "lysosomal disease". Here again, it is not clear if there is any connection with any known lysosomal disorder. If the authors mean that they unexpectedly created a model of Danon disease, they should say so, and they should explain what the advantages of this model.
Specific Comments:
The authors should clearly indicate why they chose skeletal muscle for tissue-specific deletion of Vps15.
These important points were in part clarified in our answer to the point #3 of reviewer #1. As discussed in the revised manuscript, the main goal of our manuscript was to study the mammalian pathophysiology of Vps15/Vps34 signal transduction and the putative cross-talk with mTOR. We mainly focussed on skeletal muscle because previous work from our lab and collaborators defined the impact of mTOR/S6K and Atg gene deletions in skeletal muscle. We reveal AVM in mice as a consequence of Vps15/Vps34 inactivation, a phenotype well distinct from Atg5/7 and mTOR mutants. Whether this directly applies to a real human disease will have to wait for the demonstration that components of the Vps34 signalling pathway are mutated in AVM or lysosomal storage diseases. The interest at this stage is to have defined a signal transduction pathway that causes AVM when it is defective and ameliorates the disease condition when it is up-regulated.

2) The term "Vps15-null" is misleading since a 100kDa product is made after Cre recombination. Importantly, the presence of this product is very disturbing, because this protein may well be a culprit responsible for the unexpected results. At least, one cannot rule this out.
We agree with both reviewers #2 and 3 that one needs to be cautious about the gene targeting strategy. In this revised version we performed a variety of experiments to conclude that the truncated Vps15 completely loses Class III PI3K signaling and does not acquire new functions that might explain the observed effects on mTOR signaling and lysosomal trafficking. The experimental approaches include overexpression of truncated form by adenoviral transduction and immunocomplex kinase assays after pull down with different components of Vps15 partners, as outlined in the point #1 to reviewer #2.

3) In addition, it is not clear if this truncated protein is made in skeletal muscle of muscle-specific KO mice: Figure 5A does not show any lower band in the KO. Please clarify this point. Another problem with muscle specific KO is a great variability between different mice/samples (i.e., for the Vps15 bands in Fig 5A)? Please comment more on this, beyond simply mentioning incomplete excision.
To confirm the presence of Vps15 truncated protein in Vps15-mutant muscles we reloaded the protein extracts and probed with Vps15 antibody. We are presenting the result of this analysis on the modified Fig. 5A, where we are able to detect truncated Vps15 band in Vps15 mutants muscles but not in controls. The mouse to mouse variability in these mutants is not higher than it was observed in previous studies on distinct mutants. Our conclusions in the immunoblot experiments are supported by the analysis of 3-6 mouse samples to take into account possible outliers.

4) The paper claims that there is an increase in the number of lysosomes. What is the mechanism for the stimulation of lysosomal biogenesis (Figure 6 C)? This deserves some comments. And again, what if the stimulation of the lysosomal biogenesis is a function of the 100 kDa protein? Given the increase in the number of lysosomes and autophagosomes, what is the mechanism of incomplete flux?
We thank the reviewer for raising these issues that improved our study. To address the question of increased lyososomal biogenesis we have measured by qPCR the levels of TFEB transcription factor and its targets. TFEB is a transcription factor that has been shown to coordinate expression of lysosomal hydrolases, membrane proteins and genes involved in autophagy (Settembre C et al. Science. 2011 332:1429-33.). As seen in Supporting Information Fig. 8, there is no upregulation of the TFEB transcriptional response in Vps15-depleted muscles. We therefore favor the possibility that the increased lysosomal mass is due to incomplete flux and recycling rather than increased biogenesis. To address the possible mechanisms of impaired lysosomal function, in the new Fig. 3D we assay multiple lysosomal enzymes in MEF cell extracts and in the extracellular medium. The striking increase in the extracellular versus intracellular activity ratio indicates an impairment of lysosomal enzyme sorting as a consequence of Vps15 deletion.

5) In Vps15-deficient skeletal muscle, glycogen is shown to accumulate in the cytosol and in autophagosomes. Does glycogen accumulate in lysosomes? Does deletion of Vps15 in muscle mimic Pompe disease? Is this what the reference to "lysosomal disease" in the title refers to?
The reviewer is correct that these evidences would strengthen the link with lysosomal storage disorders. In this revised version, we show a deeper analysis of electron microscopy images (new Fig. 8C, Supporting Information Fig. 10). We now show representative images of lysosomes filled with glycogen, similar to Pompe disease mouse models. Furthermore, we now compare glycogen, dystrophin and p62 staining in *Vps15*-deficient muscles with muscle samples from the mouse model of Danon disease (Lamp2 knockout mice). In Supporting Information Fig. 11B, one can appreciate the similarities between the mouse mutants.

6) In Figure 9A, the authors analyze the effect of Vps15 over-expression in Vps15-depleted MEFs. It is not clear why this over-expression would increase the amount of 100 kDa product in the Cre condition but not in the GFP (control) condition.
From these experiments, it looks like over-expression of Vps15 in Vps15-depleted MEFs does not affect the level of Vps34 but still affects p62 (Fig 9A), suggesting that Vps34 is not important for this phenomenon. If indeed Vps15 overexpression alone was sufficient to have an effect on p62, what is the reason for using transfection with Vps15 and Vps34 combined for human myoblasts (Fig 9B)? The use of the combination of Vps15 and Vps34 needs to be justified.

© EMBO

In this revised version, we have transduced Vps15-mutant cells with increasing MOI of full length Vps15 expressing adenovirus. As shown in Supporting Information Fig. 12B, the levels of truncated Vps15 remains fairly constant in mutant cells, though low amounts of full length Vps15 are sufficient to affect LC3 and p62 levels.
Concerning the Vps34 levels, the overexpression of Vps15 rescues the levels of Vps34 in MEFs. To support this we add a longer exposure for the Vps34 immunoblot in the Fig. 9B. This is also clearly seen in the new Supporting Information Fig.13A.
These data are consistent with the previous literature clearly pointing to the need of stoichiometric amounts of Vps15 and Vps34 in Class III PI3K signal transduction. Thus, overexpression of Vps15 alone is not sufficient to alleviate LC3 accumulation in Danon disease patient myotubes (Supporting Information Fig. 14A).

It looks like Vps15 is an abundant protein in MEFs and in skeletal muscle but not in human myoblasts (in either control or in Danon disease; compare Fig 5A, 9A-B). The authors need to elaborate.

As shown in the figure below, we loaded on the same gel mouse tissues (muscles and MEFs) and human myotubes. The data indicate similar Vps15 levels among the cell lines samples.

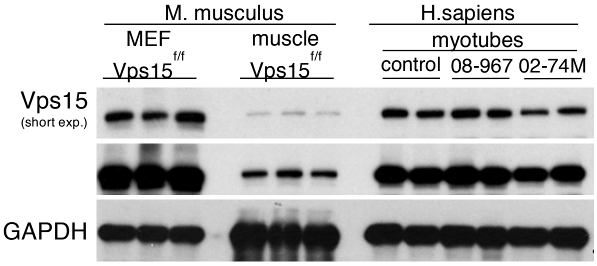

Vps15 expression in MEFs, mouse skeletal muscles and human myotubes. 10ug of total protein lysate were analyzed by immunobloting with indicated antibodies.

Minor comments:
It would be nice to provide a reference for the lysosomal storage disease incidences described on page 20.
Italics or no italics on "et al" for citations (e.g., page 3)
Page 20: The content for the reference to Atg7KO is not correct; the claim that Atg7 KO on Pompe background worsens phenotype is not accurate.

The reference "Yan and Becker, 2007" (page 7) does not seem to correspond to the role of Vps15 in stability of the complex.

Page 10: methyltransferase missing "n" (type-o)

We corrected these points in the revised version

Accepted 13 March 2013

Please find enclosed the final reports on your manuscript. We are pleased to inform you that your manuscript is accepted for publication and is now being sent to our publisher to be included in the next available issue of EMBO Molecular Medicine.

***** Reviewer's comments *****

Referee #2 (Comments on Novelty/Model System):

The manuscript has been improved with most of the issues raised by reviewers being adequately

addressed by the authors.

Referee #3 (Comments on Novelty/Model System):

The authors addressed most of my concerns and the paper is much improved
