# Supplementary material for: Defects of Vps15 in skeletal muscles lead to autophagic vacuolar myopathy and lysosomal disease
Source: EMBO Mol Med. 2013 Apr 30;5(6):870–90. doi: 10.1002/emmm.201202057 (PMC3779449; doi:10.1002/emmm.201202057)
Supplement: Supplementary file 2 [file emmm0005-0870-SD2.pdf]

Supporting Information

Defects of Vps15 in skeletal muscles lead to autophagic vacuolar myopathy and lysosomal disease

Ivan Nemazanyy, Bert Blaauw, Cecilia Paolini, Catherine Caillaud, Feliciano Protasi, Amelie Mueller, Tassula Proikas-Cezanne, Ryan C. Russell, Kun-Liang Guan, Ichizo Nishino, Marco Sandri, Mario Pende, Ganna Panasyuk

Table of contents

Supporting Information Figures 1-14

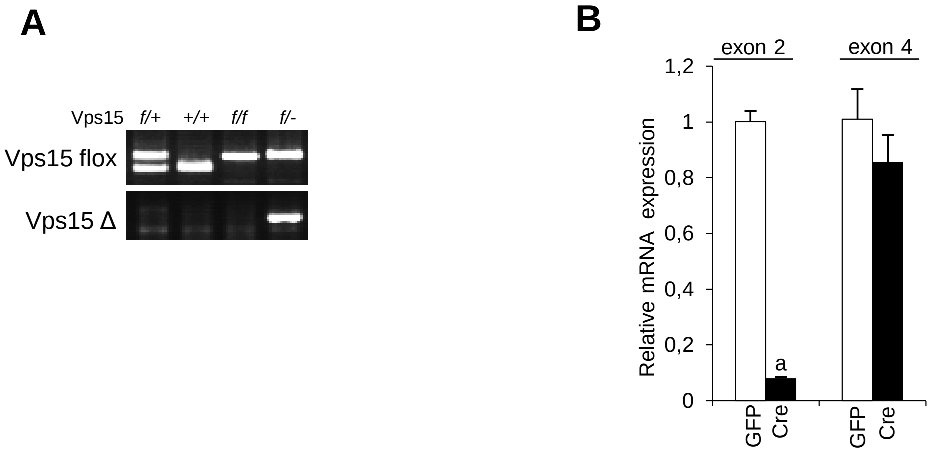

Supporting Information Figure 1. Generation of whole body *Vps15*$^{f/f}$ knockout mice

(A) Representative tail snip genotyping of the pups derived of CMV-Cre^{+};Vps15$^{f/f}$ crosses to discriminate floxed and recombined *Vps15* allele using EF/ER and EF/LR primer pairs, accordingly. **(B)** RTqPCR analysis of Vps15 expression in Vsp15$^{f/f}$ MEFs cells transduced with GFP or Cre adenoviruses. Primer pair within exon 2 is used to assess the efficiency of Cre-mediated excision and primer pair within exon 4 to control the level of endogenously expressed truncated and full-length Vps15. Data are mean ±SEM ($P<0.05$ a: vs GFP).

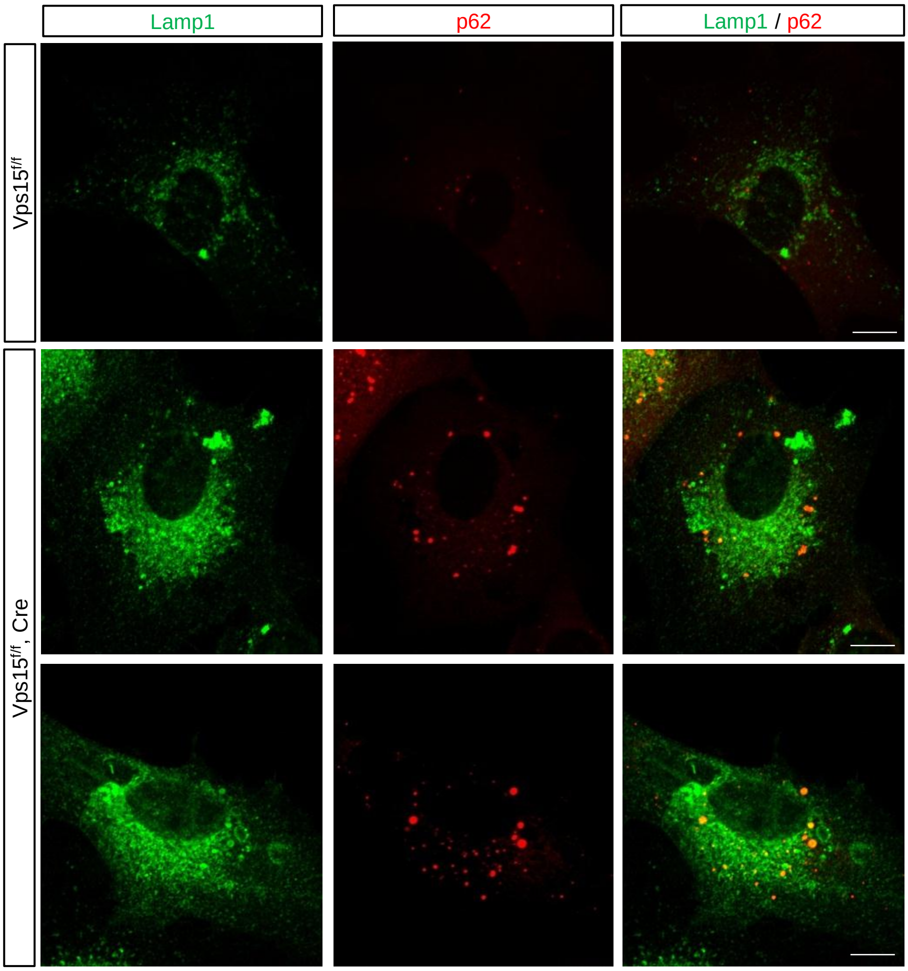

Supporting Information Figure 2.
Double-immunofluorescence staining of control and *Vps15*-depleted MEFs using anti-p62 and anti-LAMP1 antibodies. Scale bars: 10 μm.

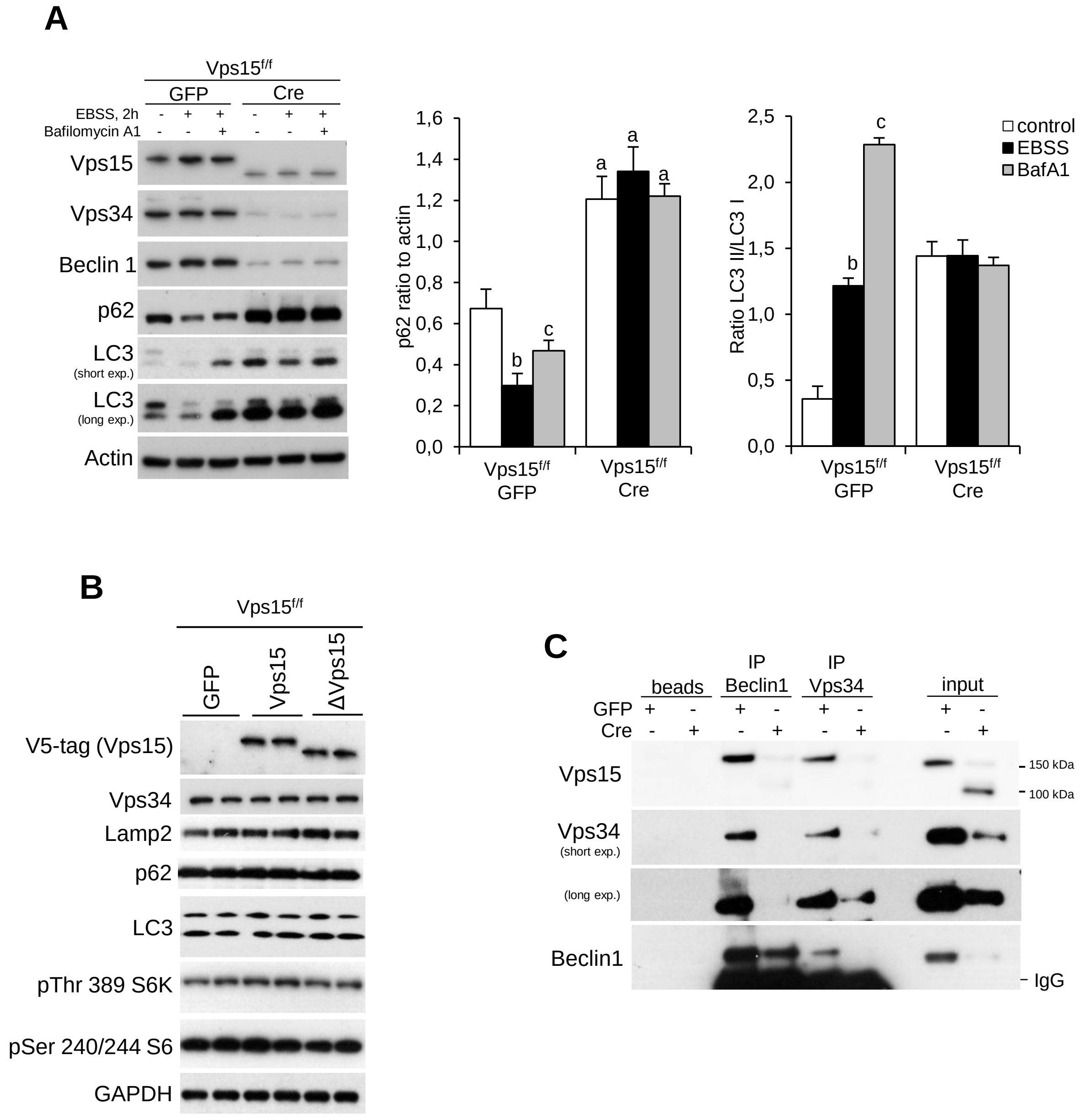

Supporting Information Figure 3.

(A) Immunoblot analysis of total protein extracts of *Vps15*-depleted MEFs treated with EBSS for 2 hours with or without 200 nM bafilomycin A1 using indicated antibodies. The ratio of p62 to actin and ratio of two LC3 forms of the densitometric assay is presented as a graph. Data are mean ±SEM (n=4, $P \leq 0.05$ a: vs GFP; b: vs control; c: vs EBSS).

(B) Expression studies of the control cells overexpressing truncated 100kDa or full-length Vps15. MEFs were transduced with indicated adenoviral vectors, collected 60 hours postinfection and total protein extracts were immunobloted with indicated antibodies. **(C)** Truncated 100kDa Vps15 does not interact with components of Class III PI3K complex. The presence of full-length Vps15 and truncated100 kDa Vps15 in the immunoprecipitates of anti-Vps34 and anti-Beclin1 antibodies was analysed from control and *Vps15*-depleted cells by immunoblotting.

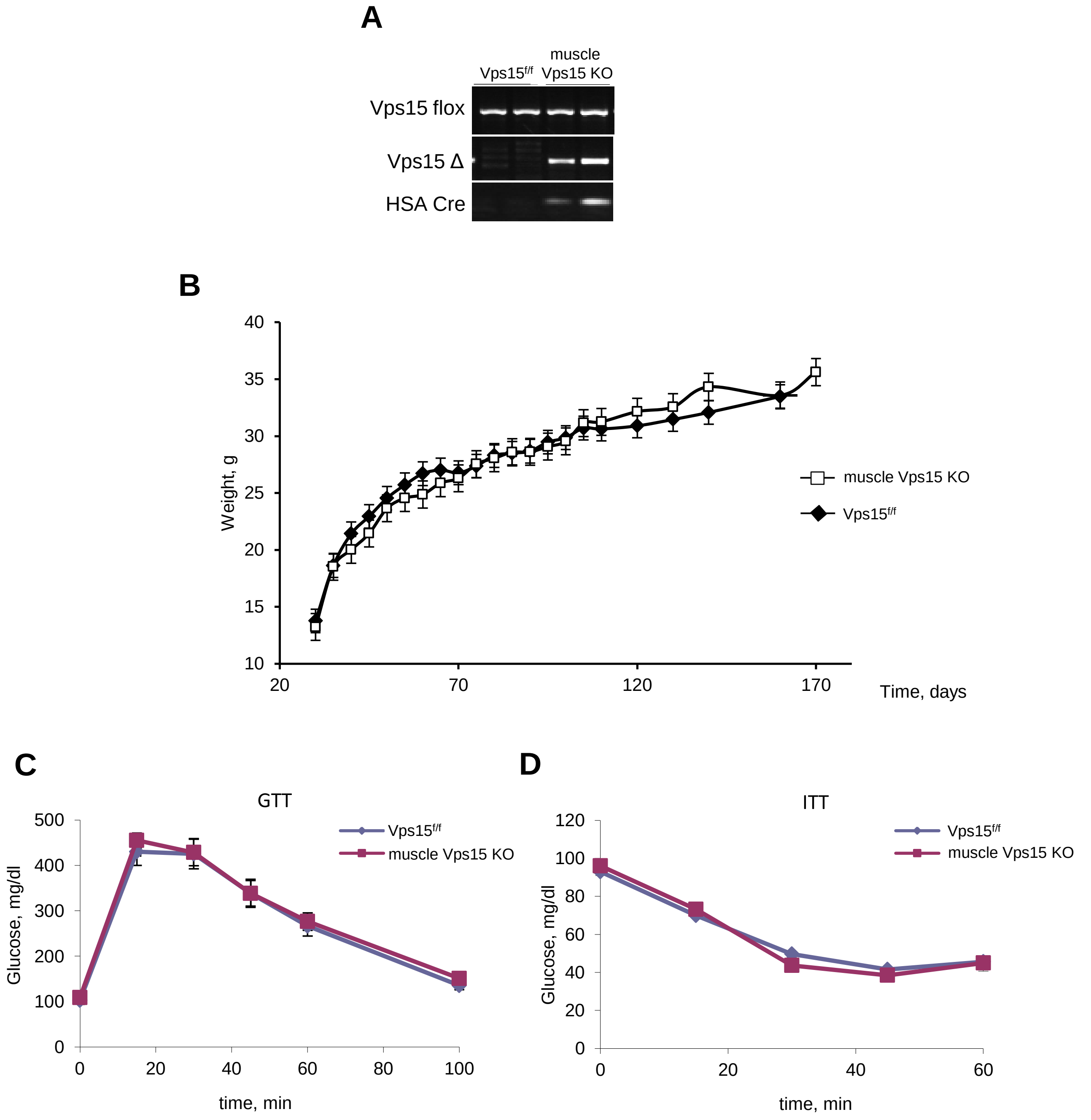

Supporting Information Figure 4. Muscle specific deletion of *Vps15* does not affect whole body glucose metabolism

(A) PCR analysis of genomic DNA from TA muscle of 2 month old muscle Vps15 KO mice and control mice using EF/ER and EF/LR primer pairs, accordingly. **(B)** Growth curves of muscle Vps15 KO mice and control mice. Data are mean ±SEM.

Glucose tolerance test (GTT) **(C)** and insulin tolerance test (ITT) **(D)** of 2 month old muscle Vps15 KO mice and control mice after intraperitoneal injection of glucose or insulin. Data are mean ±SEM.

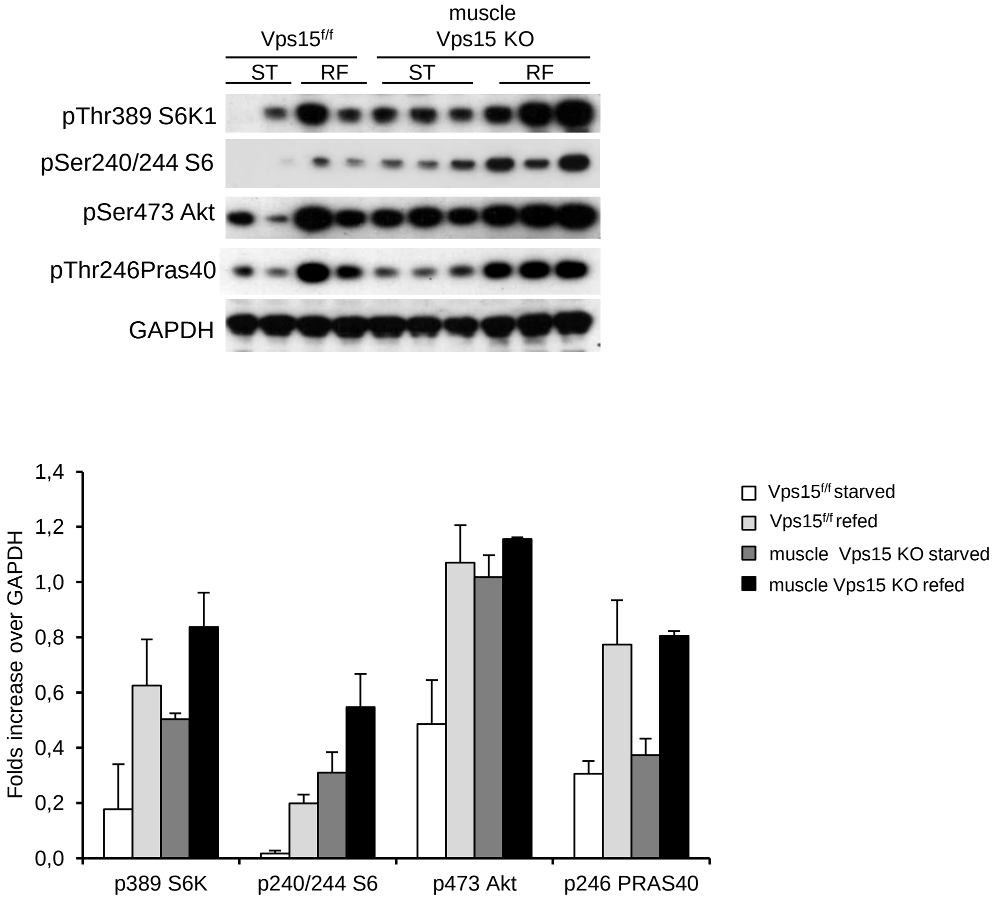

Supporting Information Figure 5. mTOR signaling in Vps15 KO muscles

Immunoblot analysis with indicated antibodies of mTORC1 and mTORC2 activity in TA muscles of 3 month old muscle Vps15 KO and control mice under starved and refed conditions. Mice were starved for 16 hours and then refed for 3 hours. The ratio of phosphorylated proteins to GAPDH of the densitometric assay is presented. Data are mean ±SEM.

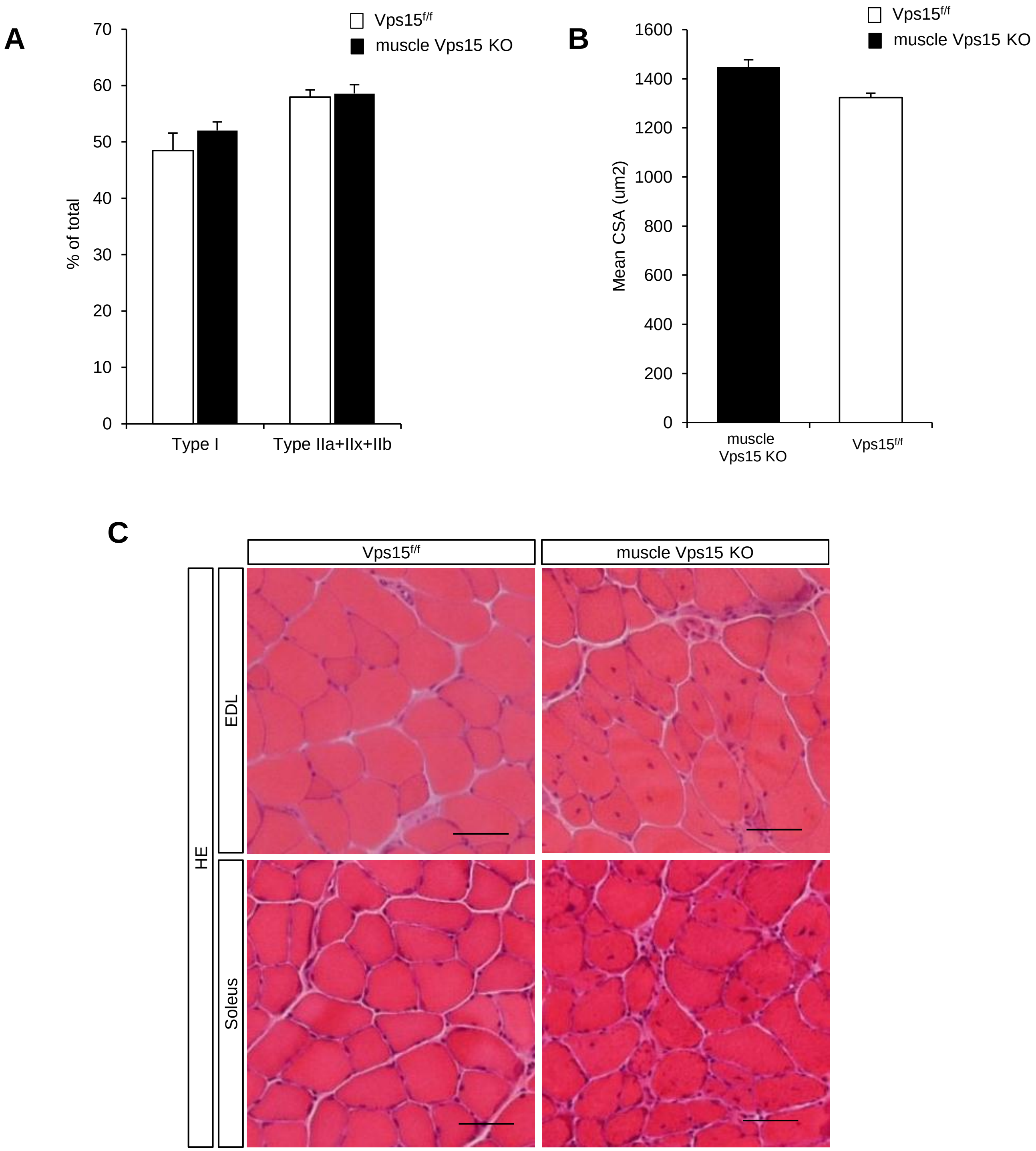

Supporting Information Figure 6. Characterization of Vps15 KO Soleus and EDL muscles

(A) Graph showing the percentage distribution of MHC isoforms I and II in Soleus muscle of 4 month old muscle Vps15 KO mice and matching controls. Data are means ±SEM. **(B)** Analysis of the mean fibre CSA in Soleus muscle of 4 month old muscle Vps15 KO mice and matching control mice. Data are means ±SEM. **(C)** Histological analysis of Soleus and EDL muscles of 2 month old Vps15 muscle KO mice and matching control mice by HE staining. Scale bars: 50 µm.

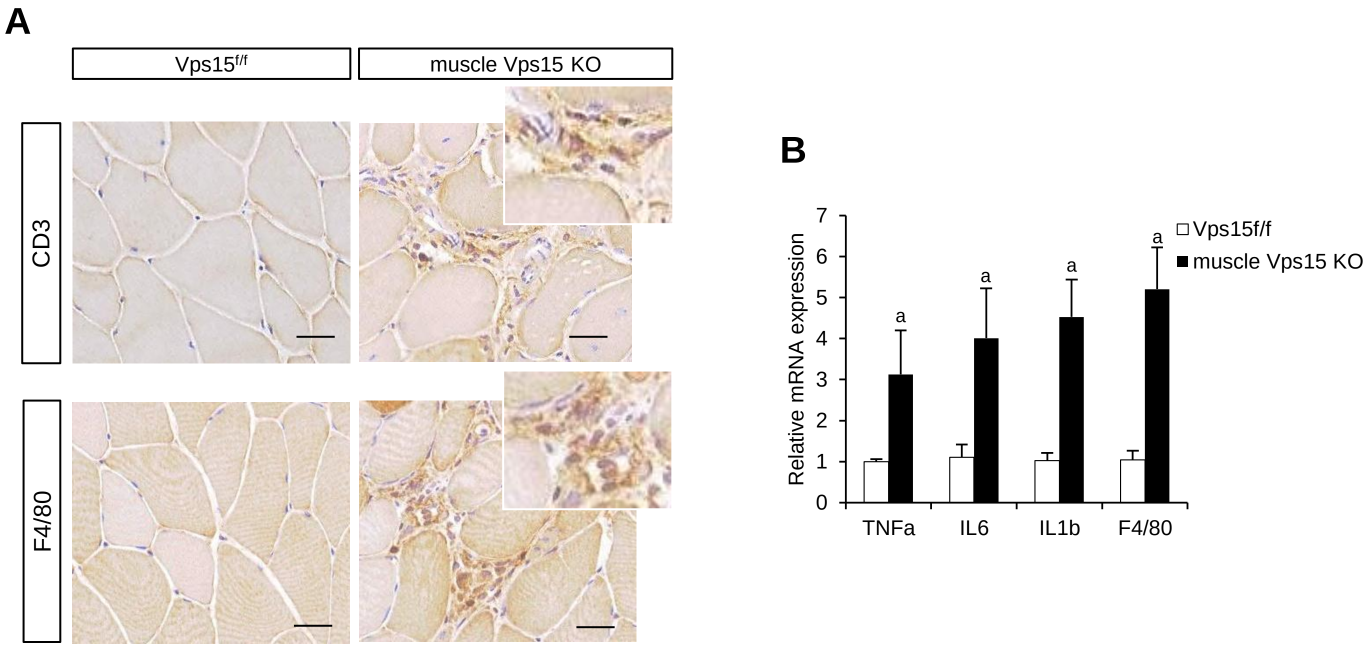

Supporting Information Figure 7. Muscle damage is accompanied by inflammatory response in Vps15 KO muscles

(A) Histological analysis of TA muscle of 4 month old Vps15 muscle KO mice and matching control mice by staining with markers of T-cells (CD3) and macrophages (F4/80). Scale bars: 40 µm. **(B)** RTqPCR analyses of the transcript levels of pro-inflammatory cytokines and immune cell markers in TA muscle of 4 month old Vps15 muscle KO and matching control mice. Data are means ±SEM (n=5, P≤0.05 a: vs Vps15$^{f/f}$).

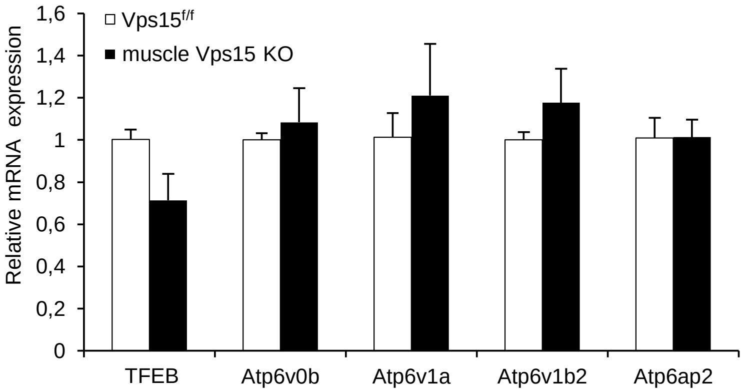

Supporting Information Figure 8.
Relative transcript levels of TFEB transcription factor and its targets in TA muscle of 4m old muscle Vps15 KO mice and matching control mice. Data are mean ±SEM.

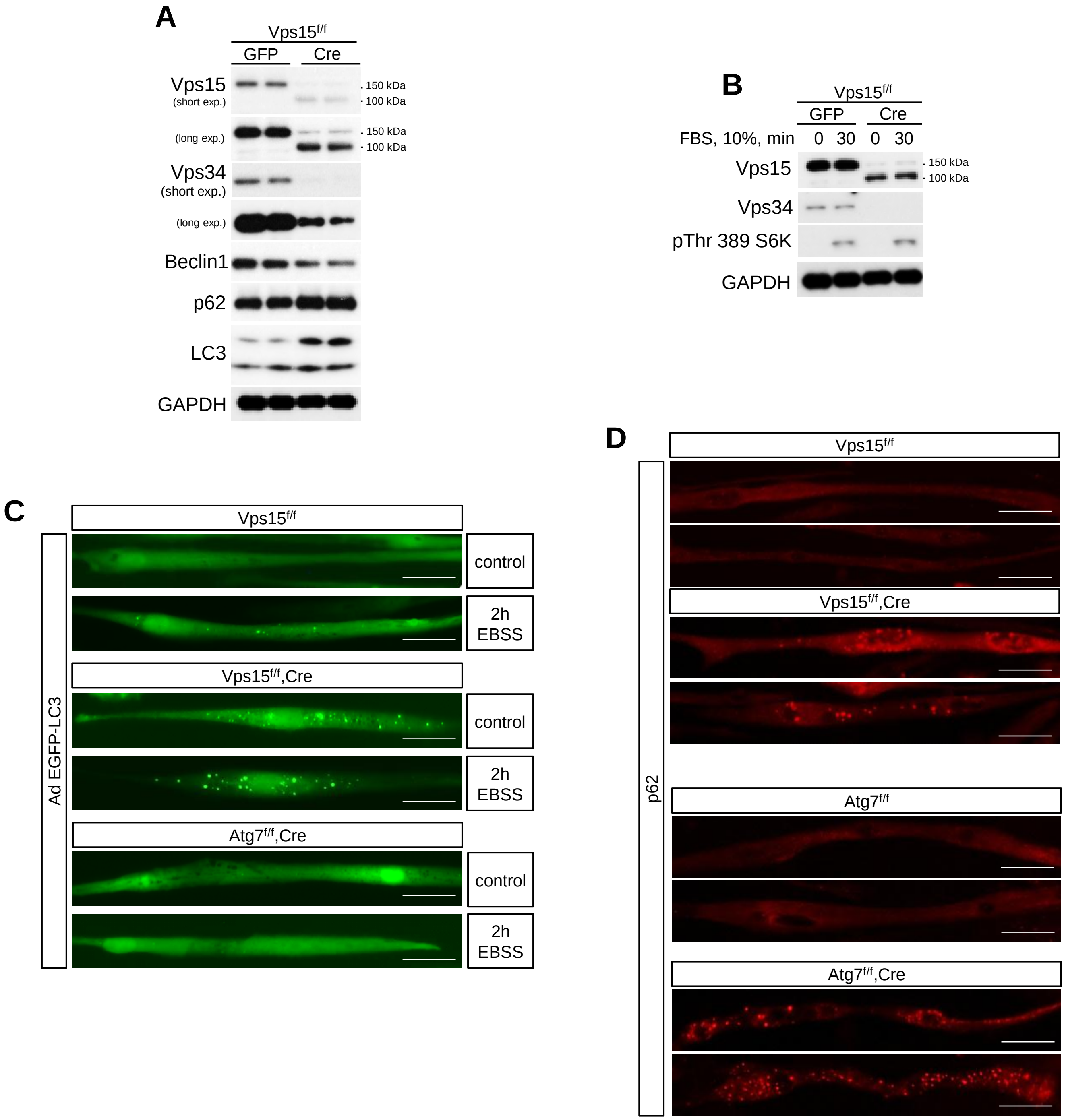

Supporting Information Figure 9. Cell autonomous effects of *Vps15* and *Atg7* depletion in primary myotubes

(A) Immunoblot analysis of *Vps15*-depleted and control myotubes with indicated antibodies. **(B)** Growth factor signaling in *Vps15*-depleted myotubes. Myotubes were nutrient starved and then stimulated with non-dialyzed FBS for 30 minutes. Total protein extracts were immunobloted with indicated antibodies. **(C)** Control, *Vps15*-depleted and *Atg7*-depleted myotubes were infected with EGFP-LC3 expressing adenoviral vector, 24 hours postinfection cells were starved for 2 hours in EBSS, fixed and analysed by immunofluorescence. **(D)** Immunofluorescence staining of control, *Vps15*-depleted and *Atg7*-depleted myotubes using anti-p62 antibody. Scale bars: 30 µm.

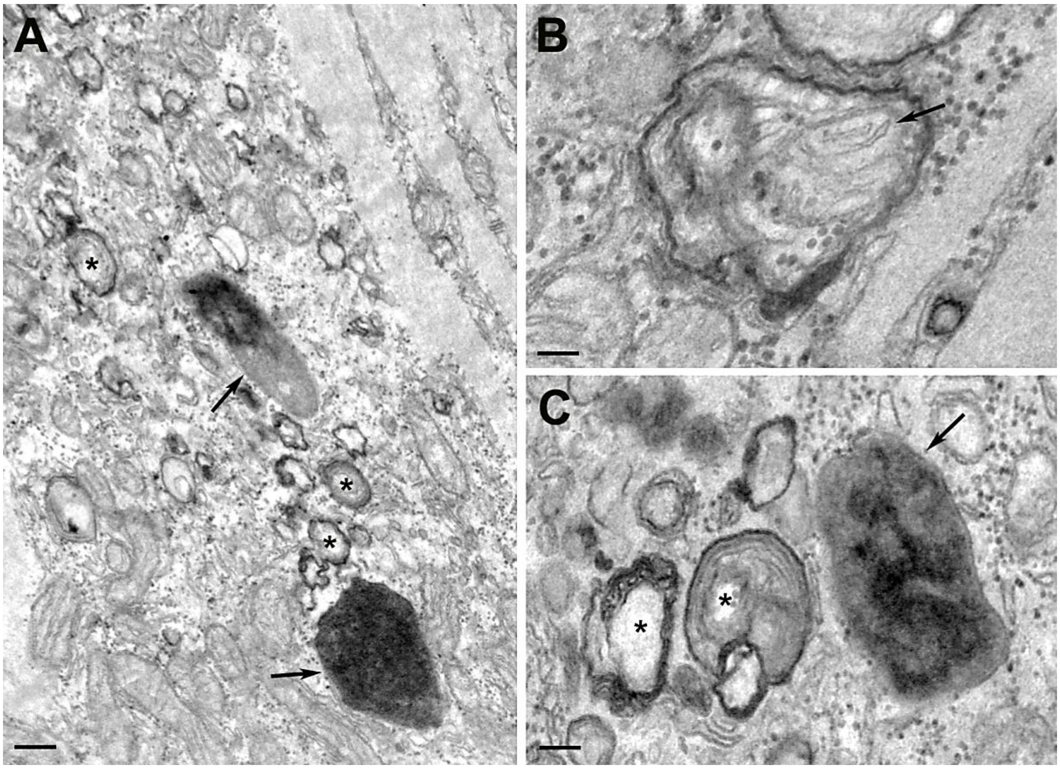

Supporting Information Figure 10. Ultrastructural analysis of *Vps15*-deficient muscles **(A)** Low magnification micrograph showing accumulation of numerous autophagic vacuoles along the fiber: lysosomes (arrows) and autophagosomes (asterisks). **(B)** Enlargement of autophagic vacuoles containing glycogen granules and enclosing a mitochondrion (arrow). **(C)** Enlargement of a large lysosomes, filled of glycogen and of two autophagic vacuoles (asterisks). Scale bars: A 0,2 µm; B and C 0,1 µm.

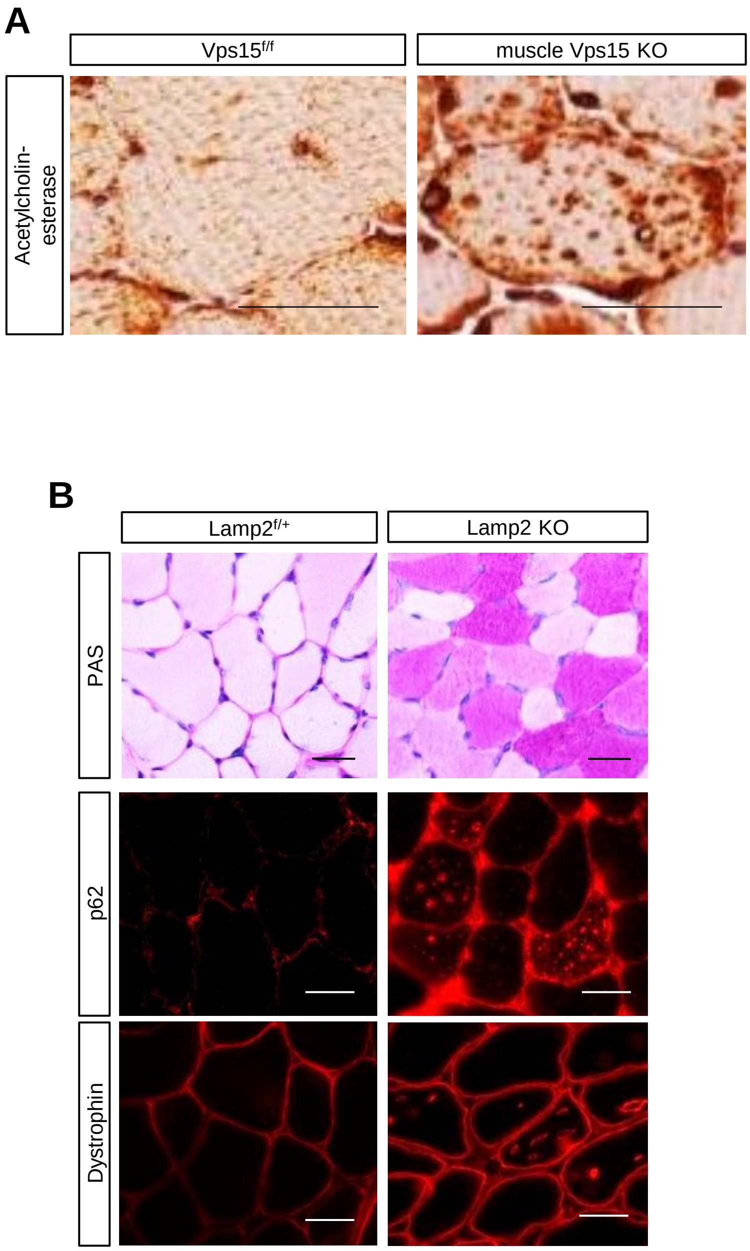

Supporting Information Figure 11.

(A) Sarcolemmal features in Vps15 KO muscles detected by Acetylcholinesterase immunostaining of TA muscles of 4 month old Vps15 muscle KO and control mice. Muscles were formalin fixed and embedded with paraffin. Scale bars: 40 µm.

(B) Histological analysis of TA muscles of 4 month old Lamp2 knockout mice and matching control mice by Periodic acid Schiff (PAS) staining to detect glycogen, and immunostaining using anti-p62 and anti-Dystrophin antibodies. Scale bars: 40 µm.

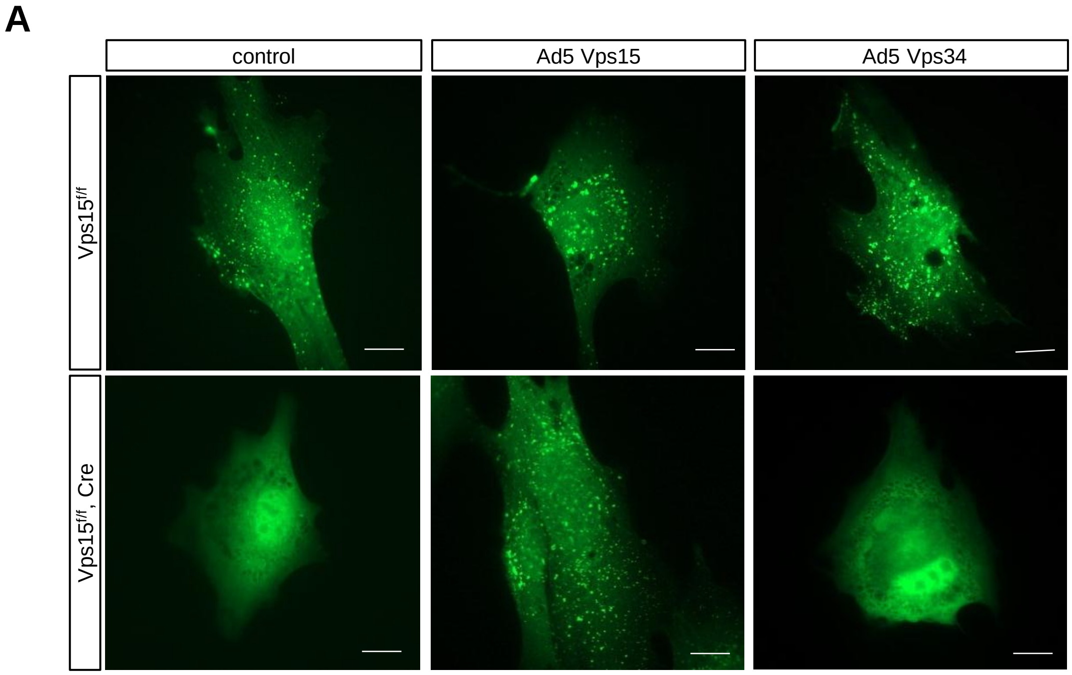

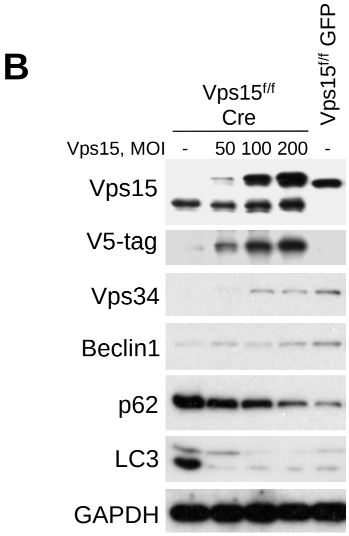

Supporting Information Figure 12. Vps15 overexpression in *Vps15*-depleted MEFs rescues PI3P levels and stimulates autophagy

(A) *Vps15*-depleted MEFs were infected with Vps15 or Vps34 adenoviruses, 36 hours postinfection cells were transfected with 2xFYVE-GFP expressing plasmid. PI3P positive compartments were visualised by confocal microscopy 24 hours posttransfection. Scale bars: 20 μm.

(B) Overexpresed Vps15 rescues the phenotype of Vps15-delpeted cells in dose dependent manner. *Vps15*-depleted MEFs were transduced with increasing concentrations of adenoviral Vps15 expressing vector, cells collected 60 hours postinfection and total protein extracts immunobloted with indicated antibodies.

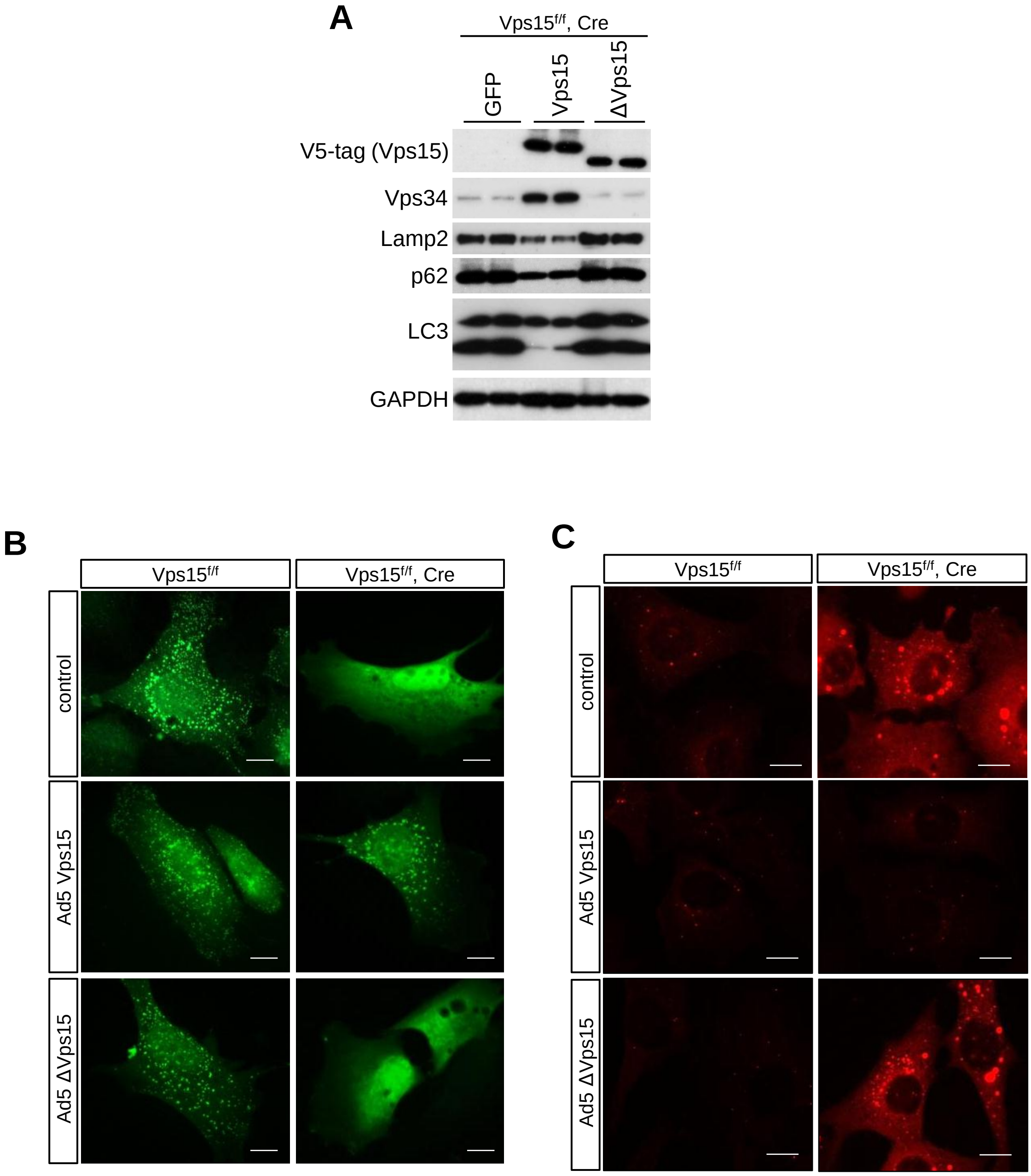

Supporting Information Figure 13. Overexpression of truncated 100kDa Vps15 cannot substitute full-length protein

(A) *Vps15*-depleted MEFs were transduced with indicated adenoviral vectors, collected 60 hours postinfection and total protein extracts were immunobloted with indicated antibodies. **(B)** PI3P positive compartments were visualised in control and *Vps15*-depleted MEFs with and without Vps15 overexpression. Cells were transfected with 2xFYVE-GFP expressing plasmid. 36 hours post transfection cells were fixed and PI3P positive compartments were revealed by confocal microscopy. **(C)** Immunofluorescence staining of control and *Vps15*-depleted MEFs with and without Vps15 overexpression using anti-p62 antibodies. Scale bars: 20 µm.

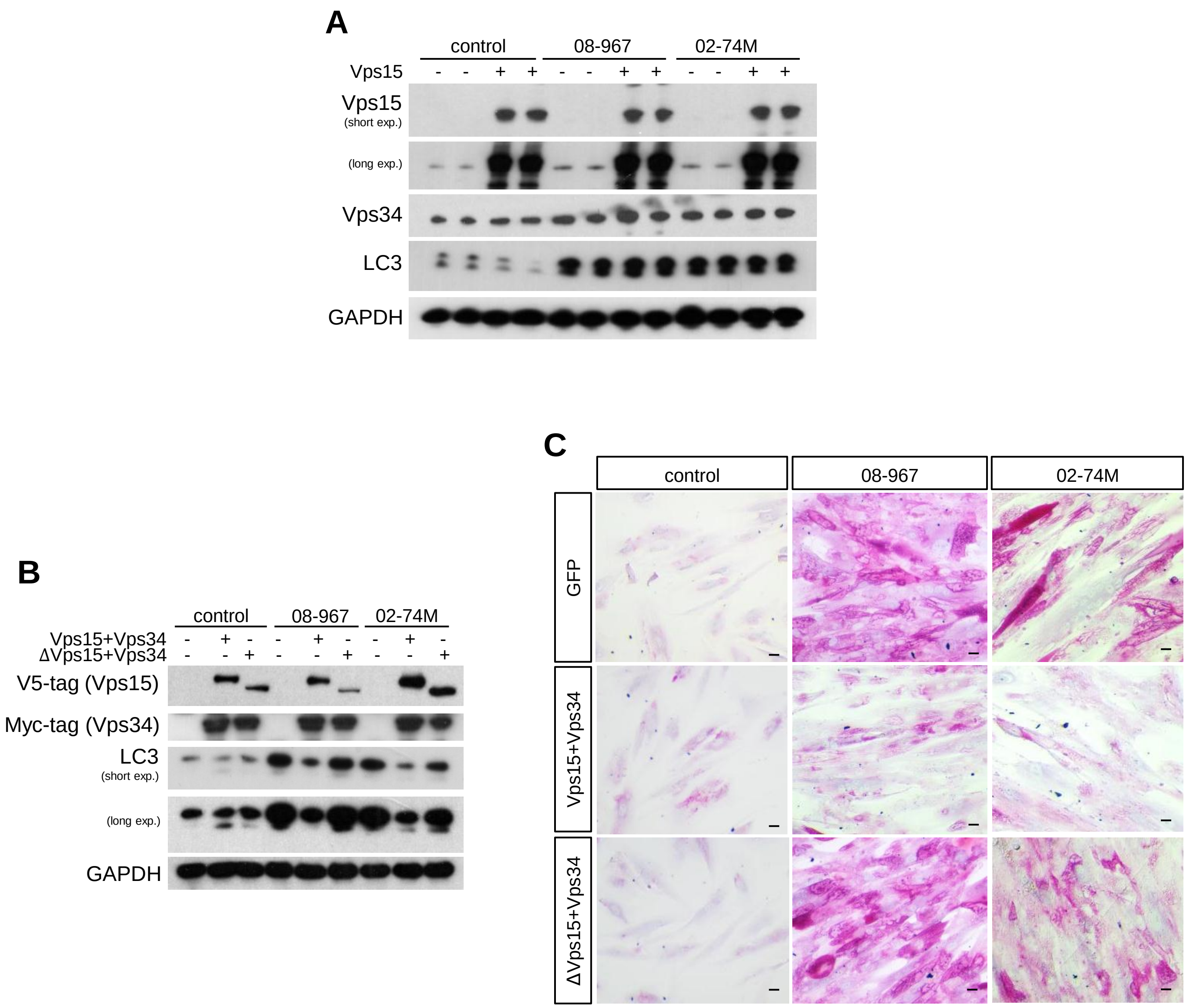

Supporting Information Figure 14.

(A) Overexpression of Vps15 alone in primary myotube cultures from Danon disease patients does not upregulate autophagy. Human myoblasts derived from Danon disease patients were differentiated to myotubes and transduced 2 days postdifferentiation with adenoviral vector overexpressing full-length Vps15. Cells were collected 3 days postinfection and total protein extracts analysed by immunoblotting with indicated antibodies.

(B) Overexpression of truncated Vps15 in primary myotube cultures from Danon disease patients does not affect autophagy. Human myoblasts derived from Danon disease patients were differentiated to myotubes and transduced 2 days postdifferentiation with Vps15 and Vps34 adenoviral vectors. Cells were collected 3 days postinfection and total protein extracts were analysed by immunoblotting with indicated antibodies. **(C)** PAS staining of the glycogen in human myotubes derived from Danon disease patients transduced with GFP, Vps15/Vps34 or truncated 100kDa Vps15/Vps34 adenoviral vectors, analysed 3 days postinfection. Scale bars: 20 μm.
